# GBF3 transcription factor imparts drought tolerance in *Arabidopsis thaliana*

**DOI:** 10.1038/s41598-017-09542-1

**Published:** 2017-08-22

**Authors:** Venkategowda Ramegowda, Upinder Singh Gill, Palaiyur Nanjappan Sivalingam, Aarti Gupta, Chirag Gupta, Geetha Govind, Karaba N. Nataraja, Andy Pereira, Makarla Udayakumar, Kirankumar S. Mysore, Muthappa Senthil-Kumar

**Affiliations:** 1 Department of Crop Physiology, University of Agricultural Sciences, Bengaluru, 560065 India; 2 Department of Crop, Soil and Environmental Sciences, University of Arkansas, Fayetteville, USA; 3 Noble Research Institute, 2510 Sam Noble Parkway, Ardmore, OK 73401 USA; 4 ICAR-Central Institute for Arid Horticulture, Indian Council of Agricultural Research, Bikaner, 334006 India; 5 National Institute of Plant Genome Research, Aruna Asaf Ali Marg, PO Box No. 10531, New Delhi, 110 067 India; 6 ICAR-National Institute of Biotic Stress Management, Present Address: Indian Council of Agricultural Research, Raipur, 493225 India

## Abstract

Drought transcriptome analysis of finger millet (*Eleusine coracana*) by cDNA subtraction identified drought responsive genes that have a potential role in drought tolerance. Through virus-induced gene silencing (VIGS) in a related crop species, maize (*Zea mays*), several genes, including a *G-BOX BINDING FACTOR 3* (*GBF3*) were identified as candidate drought stress response genes and the role of *GBF3* in drought tolerance was studied in *Arabidopsis thaliana*. Overexpression of both *EcGBF3* and *AtGBF3* in *A. thaliana* resulted in improved tolerance to osmotic stress, salinity and drought stress in addition to conferring insensitivity to ABA. Conversely, loss of function of this gene increased the sensitivity of *A. thaliana* plants to drought stress. *EcGBF3* transgenic *A. thaliana* results also suggest that drought tolerance of sensitive plants can be improved by transferring genes from far related crops like finger millet. Our results demonstrate the role of *GBF3* in imparting drought tolerance in *A. thaliana* and indicate the conserved role of this gene in drought and other abiotic stress tolerance in several plant species.

## Introduction

Drought stress is one of the most prevalent environmental factors limiting crop productivity^1^. Plants have evolved to endure water limited conditions using an array of morpho-physiological and biochemical adaptations through activation of a cascade of molecular networks^2, 3^. Under field condition, crop plants are subjected to short-term water deficits of several days to weeks and some plant species have evolved to quickly limit the cellular damage and continue to grow in the stressful environment.

Crop plants such as pearl millet (*Pennisetum glaucum*), horsegram (*Macrotyloma uniflorum*), peanut (*Arachis hypogaea*), pigeon pea (*Cajanus cajan*) and sorghum (*Sorghum bicolor*) have been used to identify traits and genes that contribute for yield protection under drought^4–11^. Finger millet (*Eleusine coracana*) is another important crop grown in the semi-arid tropical regions of Africa and Indian sub-continent that have unpredictable weather, limited and erratic rainfall and nutrient-poor soils^12^. Finger millet has been shown to tolerate dry spells in the early stages of growth and then grow rapidly upon rewetting^13^. Finger millet exhibits higher seedling survival and leaf area retention under drought stress compared to sunflower (*Helianthus annuus*), cowpea (*Vigna unguiculata*), beans (*Phaseolus vulgaris*) and tomato (*Solanum lycopersicum*)^7^. Yield protection in this crop has been shown to be better than other crops during drought^14^. Therefore, in this study we attempted to profile the transcriptome of finger millet under drought stress.

Plant adaptation to drought stress has been shown to be orchestrated by regulated expression of several stress responsive genes^15^. The products of these genes are directly involved in cellular protection from stress-damage (e.g. osmoprotectants, antioxidants and chaperons), signal transduction and transcriptional control^15–18^. Several genes from these categories are known to be regulated by transcription factors by binding to the specific promoter elements and regulate their expression^19^. The role of several transcription factors in regulating drought tolerance is well understood^20^. For example, overexpression of members of bZIP, APETELA2 (AP2)/ERF, NAC, zinc-finger, HSF, MYB, bHLH (MYC) and WRKY super family transcription factors has resulted in improved tolerance of plants to drought stress through coordinated regulation of genes involved in cellular protection and stress adaptation^17, 21–29^. In this study, we identified early drought stress induced genes from finger millet by subtractive hybridization. Transcript expression pattern was further confirmed by quantitative RT-PCR (RT-qPCR) analysis in finger millet. Preliminary function analysis by virus-induced gene silencing (VIGS) of selected finger millet orthologs in maize (*Zea mays*) identified *Rho GTPase activating protein 2* (*RGAP2*), *DEAD/DEAH box helicase* (*DBH*), *G-box binding factor 3* (*GBF3*), *RNase S-like protein precursor* (*RSLP*), *unknown protein* (*UN*) and *hypothetical protein* (*HYP*) genes as candidate genes for studying their role in drought stress response. Furthermore, overexpression of *EcGBF3* or *AtGBF3* transcription factor improved tolerance of *Arabidopsis thaliana* (hereafter referred as Arabidopsis) plants to osmotic stress, drought and salinity in addition to showing insensitivity to ABA. Further, the *EcGBF3* overexpression imparted tolerance as like that of *AtGBF3* overexpression in drought sensitive Arabidopsis. Results from three plant species studied here indicated that GBF3, a transcription factor that potentially regulates genes encoding ABI five binding proteins, play a role in imparting drought tolerance.

## Results

### Identification of drought stress inducible genes in finger millet

**Figure 1:**
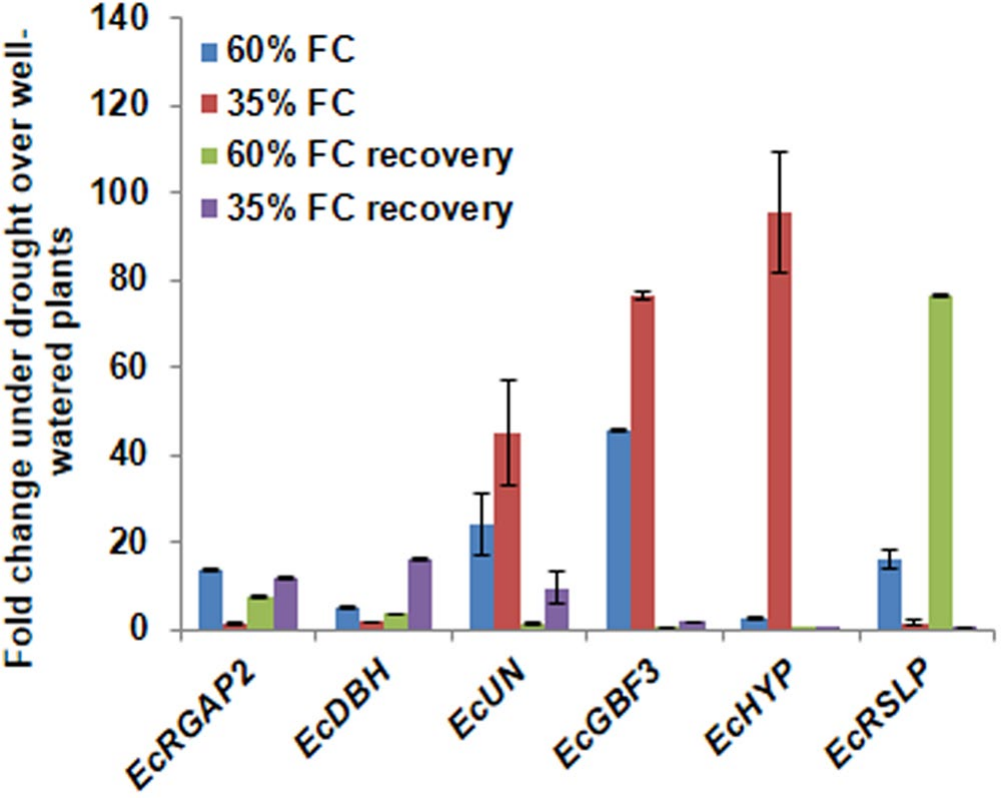
Expression of finger millet genes under drought stress and recovery. Total RNA from leaf samples subjected to different drought stress levels and recovery were used for RT-qPCR analysis. The data was normalized to *EcActin* expression levels and the relative change over control plants was calculated using 2^−ΔΔCt^ method. The values are mean ± SE of two biological replicates performed in two different instruments.

A cDNA library was constructed by subtracting control sample transcripts from drought sample transcripts (Supplementary Methods). RNA pooled from 80% field capacity (FC), 60% FC and 35% FC was used as drought sample and 100% FC as control. From the library, 156 clones were sequenced and annotated using NCBI BLASTX database and submitted to NCBI dbEST database. Among the sequenced clones, there were 139 unique expressed sequence tags (ESTs) (Supplementary Table S3). Based on the putative functions ESTs were classified into four broad functional categories (Supplementary Fig. S4). The maximum number (45%) of ESTs showed functional similarity to genes related to growth, metabolism and transport. About 23% of ESTs showed functions similar to genes involved in cellular protection. Importantly, there were 17% and 15% ESTs with predicted regulatory roles and unknown functions, respectively (Supplementary Fig. S4). Transcript expression of the selected 89 ESTs was confirmed by RT-qPCR analysis using RNA from finger millet plants subjected to different drought stress levels and recovery (Supplementary Methods). Under moderate drought stress of 60% FC, 16 ESTs showed more than 2-fold increase in transcript levels compared to control (Supplementary Fig. S5; Supplementary Table S4). These included ESTs for regulatory genes like *Rho GTPase activating protein 2* (*RGAP2;* FD661844), *Salt-stress inducible bZIP protein* (FD661884), *G-box binding factor 3* (*GBF3*; FD661896), and *RNase S-like protein precursor* (*RSLP;* FD661909), and a gene encoding unknown protein (UN; FD661919) with fold increase values of 14, 28, 46, 16 and 13, respectively. The maximum induction of ESTs was observed under severe drought stress of 35% FC with 47 ESTs showing more than 2-fold increase in transcript levels compared to control. ESTs for functional genes such as *Early light-inducible protein* (FD661789) and *Responsive to abscisic acid 17* (FD661810) showed more than 100-fold increase in transcript levels both under moderate and severe drought stress compared to control. In plants recovering from moderate drought stress of 60% FC, 15 ESTs showed greater than 2-fold increase in transcript levels. Among these, ESTs for regulatory genes *RGAP2* and *RSLP* showed 7- and 74-fold increase in transcript levels, respectively, during recovery when compared to control. In plants recovering from severe drought stress of 35% FC, 17 genes showed more than 2-fold increase in transcript levels. However, *Acyl CoA binding protein* (FD661802), *60 S ribosomal protein L10* (FD661845), *Putative Acyl-CoA binding protein* (FD661883), *Nuclear antigen like* (FD661900), *Geranyl geranyl hydrogenase* (FD661908) and *Hypothetical protein OsI_023315* (FD661916) genes were specifically induced under recovery. On the other hand, genes such as *Chlorophyll a/b binding protein* (FD661801), *Fructose-bisphosphate aldolase* (FD661912), *Enolase* (FD661852) and *Pyruvate phosphate dikinase* (FD661923) showed mild expression indicating disturbance in photosynthesis and down-regulation of carbon metabolism during drought stress. For further characterization, ESTs for regulatory genes *RGAP2*, *DEAD/DEAH box helicase* (*DBH*, FD661880), *GBF3*, and *RSLP* and two ESTs for genes encoding proteins of *UN* and hypothetical protein (*HYP;* FD661886) were selected. Transcript profile of these six ESTs under different drought stress levels and recovery is shown in Fig. 1.

### Reverse genetics-based functional analysis by virus-induced gene silencing (VIGS) to screen candidate genes identified from finger millet

So far, VIGS protocol has not been developed for finger millet. Our attempts to develop the protocol using *Brome mosaic virus* (BMV) and *Barley stripe mosaic virus* (BSMV)-based VIGS vectors are not successful yet. Therefore, we performed VIGS in maize for the orthologs of finger millet drought stress responsive genes. BMV-based VIGS system has been shown to efficiently silence genes in maize^30^ (Supplementary Fig. S6). Finger millet orthologs of maize gene fragments namely *ZmRGAP2*, *ZmDBH*, *ZmGBF3*, *ZmRSLP*, *ZmUN* and *ZmHYP* were cloned into *pBMV* vector and these constructs along with marker gene *ZmUBI7* (when silenced shows visible phenotypes such as stunting and cell death) were used for gene silencing in maize. At 14 dpi *ZmUBI7* gene silenced plants were dead due to cell death (Supplementary Fig. S7). Based on this observation all VIGS plants with normal phenotype were analysed at 14 dpi. At 14 dpi, silenced plants were subjected to drought stress by withholding irrigation for five days to quantify the endogenous transcript expression of silenced genes. Transcript levels of all the six genes studied were induced under drought stress in wild-type (WT) and vector (BMV) alone inoculated plants compared to well-watered plants (Supplementary Fig. S8). These genes were expressed at the same level both in WT and vector control plants indicating that the virus infection did not affect expression of these genes. In silenced plants, 3 to 5-fold reduction in endogenous transcript levels was observed compared to vector alone inoculated plants. These results indicated that all the selected maize genes are down-regulated in VIGS construct inoculated plants.

**Figure 2:**
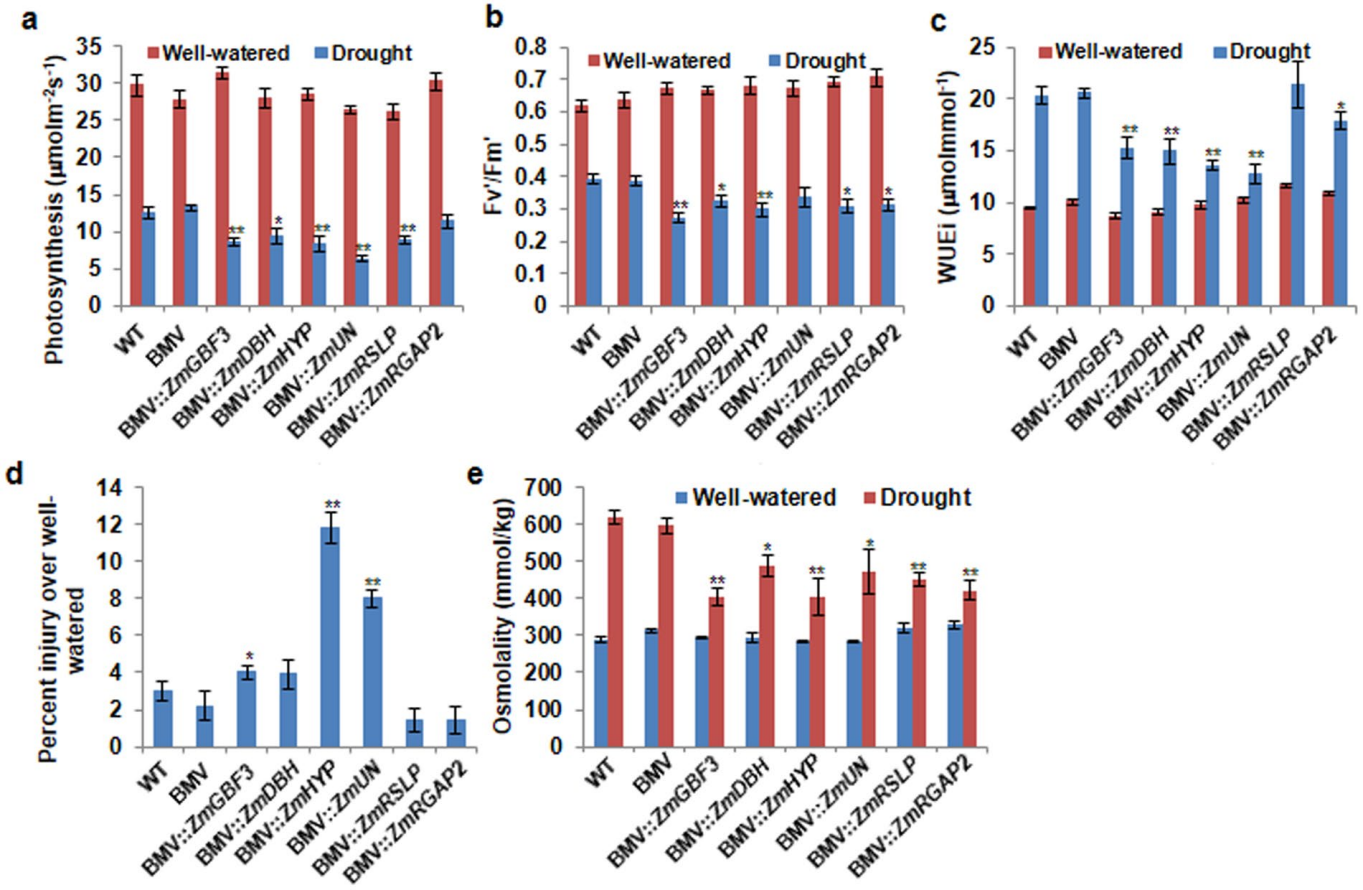
Reduced physiological performance and cellular tolerance of gene silenced maize plants to drought stress. Five plants at 14 dpi were subjected to drought stress and stress response was measured on fifth day. Photosynthesis rate (**a**), Efficiency of PSII in light adapted leaves (**b**), and Instantaneous water use efficiency (WUEi) (**c**) were measured using portable photosynthesis system. Cell membrane stability (**d**) and Osmolality (**e**) were measured from the leaf tissue. Cell membrane damage was calculated as per cent injury. BMV-inoculated plants were used as reference for statistical analysis. The values are means ± SE (n = 5). * and ** indicate significant difference from BMV-inoculated plants at *P* ≤ 0.05 and *P* ≤ 0.01, respectively. WT, non-inoculated; BMV, vector inoculated; all others with BMV:: followed by gene name are respective gene-silenced plants.

At 14 dpi, drought stress was applied to *ZmRGAP2*-, *ZmDBH*-, *ZmGBF3*-, *ZmRSLP*-, *ZmUN*- and *ZmHYP*-gene silenced and vector control plants by withholding irrigation for five days. Photochemical efficiency of PSII in a light-adapted state (Fv′/Fm′), CO_2_ assimilation (photosynthesis), transpiration and instantaneous water use efficiency (WUEi) were measured in these plants. Both under drought stress and well-watered conditions, the vector control and WT plants showed similar responses for these parameters (Fig. 2). Silencing of *ZmDBH*-, *ZmGBF3*-, *ZmRSLP*-, *ZmUN*- and *ZmHYP*- showed relative reduction in photosynthesis under drought stress compared to vector control plants (Fig. 2a). The Fv′/Fm′ was also seems to be low in the *ZmRGAP2*-, *ZmDBH*-, *ZmGBF3*-, *ZmRSLP*- and *ZmHYP*-silenced plants under drought stress (Fig. 2b). The WUEi measurements indicated a relative reduction in all gene-silenced plants compared to drought stressed vector control and WT plants (Fig. 2c).

The cellular protection of silenced plants under drought stress was examined by measuring the cell membrane integrity and osmolality over similar plants grown under well-watered conditions. The *ZmHYP*-, *ZmUN* and *ZmGBF3*-silenced plants showed relatively higher cell membrane damage as compared to vector control plants (Fig. 2d). Further, all gene-silenced plants showed significantly lower osmolality (Fig. 2e). However, reduction in relative water content under drought was found only in *ZmGBF3*-, *ZmDBH*-, *ZmHYP-* and *ZmUN*-silenced plants (Supplementary Fig. S9). Though all gene-silenced plants showed potential drought stress response, only *ZmGBF3*-silenced plants showed consistently reduced physiological performance and cellular protection under drought stress. Therefore, *GBF3* gene was chosen for further characterization in Arabidopsis.

### Characterization of *EcGBF3* and *AtGBF3* in Arabidopsis

EST sequence of *GBF3* from finger millet was used to clone the full-length cDNA (Supplementary Methods). Full-length sequence of *EcGBF3* was 1577 bp with 1086 bp coding sequence flanked by 199 bp and 292 bp 5′ and 3′ un-translated regions, respectively (GenBank accession number EU439938). The deduced 361 amino acid sequence of the coding region showed 72%, 54%, 36% and 31% identity with the maize, rice, Arabidopsis and tobacco (*Nicotiana tabacum*) *GBF3* protein sequences, respectively. Phylogenetic analysis of EcGBF3 with its Arabidopsis and maize orthologs revealed a relative closeness of EcGBF3 to ZmGBF3 (Supplementary Fig. S10a). In addition, structural motifs prediction through the MEME program showed conservation of all the motifs between EcGBF3 and ZmGBF3 in the same order (Supplementary Fig. S10b). Motif 7 and 10 are present in both EcGBF3 and ZmGBF3 but not in AtGBF3 (Supplementary Fig. S10c). These results suggest that *GBF3* gene is evolutionarily conserved between finger millet and maize, both being C4 monocots. RT-qPCR analysis using drought stressed Arabidopsis plants showed 4.5-fold increase in transcript levels of *AtGBF3* at wilting stage compared to well-watered plants, suggesting the drought induction of *AtGBF3* in Arabidopsis (Supplementary Fig. S11a). Therefore, we wanted to characterize *AtGBF3* by knock-out and overexpression analysis in Arabidopsis. In Arabidopsis, mutant lines (SALK_082840 and SALK_067963) were procured from the Salk Institute mutant collection (http://signal.salk.edu/cgi-bin/tdnaexpress). To examine the expression of *AtGBF3* in the homozygous mutant plants, two-week old mutant and wild-type (WT, Col-0) plants were subjected to drought stress by withholding irrigation until wilting. RT-qPCR analysis confirmed the loss-of-function of *AtGBF3* in SALK_082840 line and SALK_067963 line behaved like an activation line (Supplementary Fig. S11b,c). We tested the drought stress response of *EcGBF3* in comparison to *AtGBF3* by overexpression in Arabidopsis under CaMV35S promoter. In addition, the *Atgbf3* mutant background was also used to overexpress *EcGBF3* (*mEcGBF3*) and *AtGBF3* (*mAtGBF3*). Expression analysis by RT-qPCR in T_1_ generation plants selected on hygromycin indicated more than 10-fold expression of transgene in all the lines (Supplementary Fig. S12). To confirm whether activation line, SALK_067963, has overexpression phenotype we tested both SALK lines and complementation line, mAtGBF3-1, for their germination response to ABA and mannitol. Both complementation line and activation line showed increased germination compared to loss-of-function under both ABA and mannitol treatments (Fig. S13a and b). For subsequent experiments only SALK_082840 line, designated as *Atgbf3*, with T-DNA insertion in the tenth exon was used.

### Response of *GBF3* gain-of-function and loss-of-function plants to osmotic stress, salinity and ABA treatment

**Figure 3:**
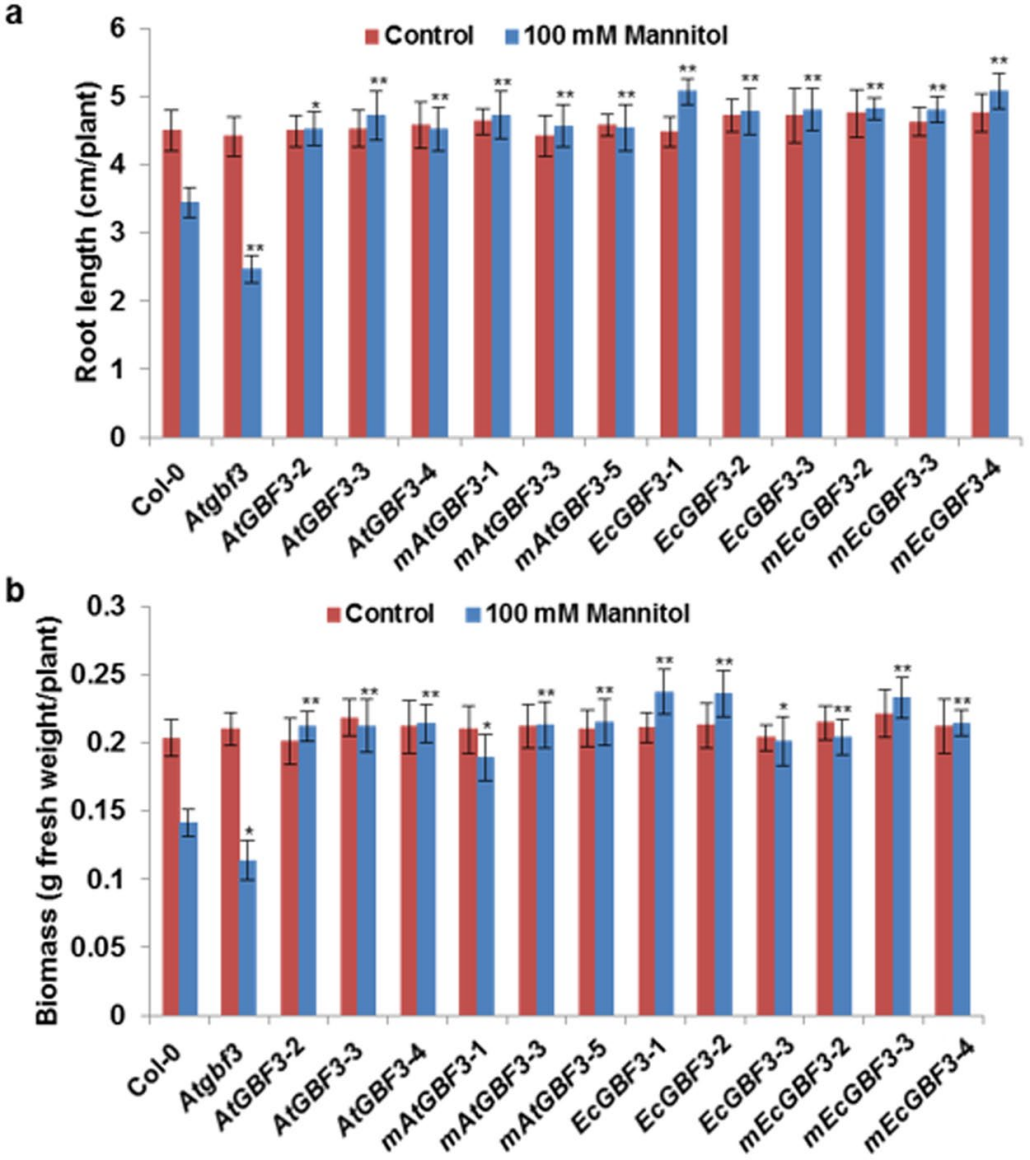
Improved osmotic stress tolerance of *AtGBF3* and *EcGBF3* transgenic Arabidopsis plants. A week old hygromycin resistant T_3_ transgenic seedlings along with Col-0 and *Atgbf3* mutant seedlings were transferred on to medium (1/2 MS, 0.5% sucrose and 0.1% phytagel) containing 100 mM mannitol and growth performance was measured after 10 days. Root length (**a**) and Biomass (**b**) were measured using plants carefully uprooted from the media and blotted on paper towel to remove any media. The values are means ± SE (n = 10). *t* test was used to show the statistical significance of the differences in the means of *Atgbf3* mutant or transgenic plants from Col-0 plants. * and ** indicate significant difference from Col-0 plants at *P* ≤ 0.05 and *P* ≤ 0.01, respectively. *Atgbf3*, *AtGBF3* mutant; *AtGBF3*-2, -3 and -4, transgenic lines overexpressing *AtGBF3*; *mAtGBF3*-1, 3 and -5, transgenic lines overexpressing *AtGBF3* in the *Atgbf3* mutant background; *EcGBF3*-1, 2 and 3, transgenic lines overexpressing *EcGBF3*; *mEcGBF3*-2, 3 and 4, transgenic lines overexpressing *EcGBF3* in the *Atgbf3* mutant background.

**Figure 4:**
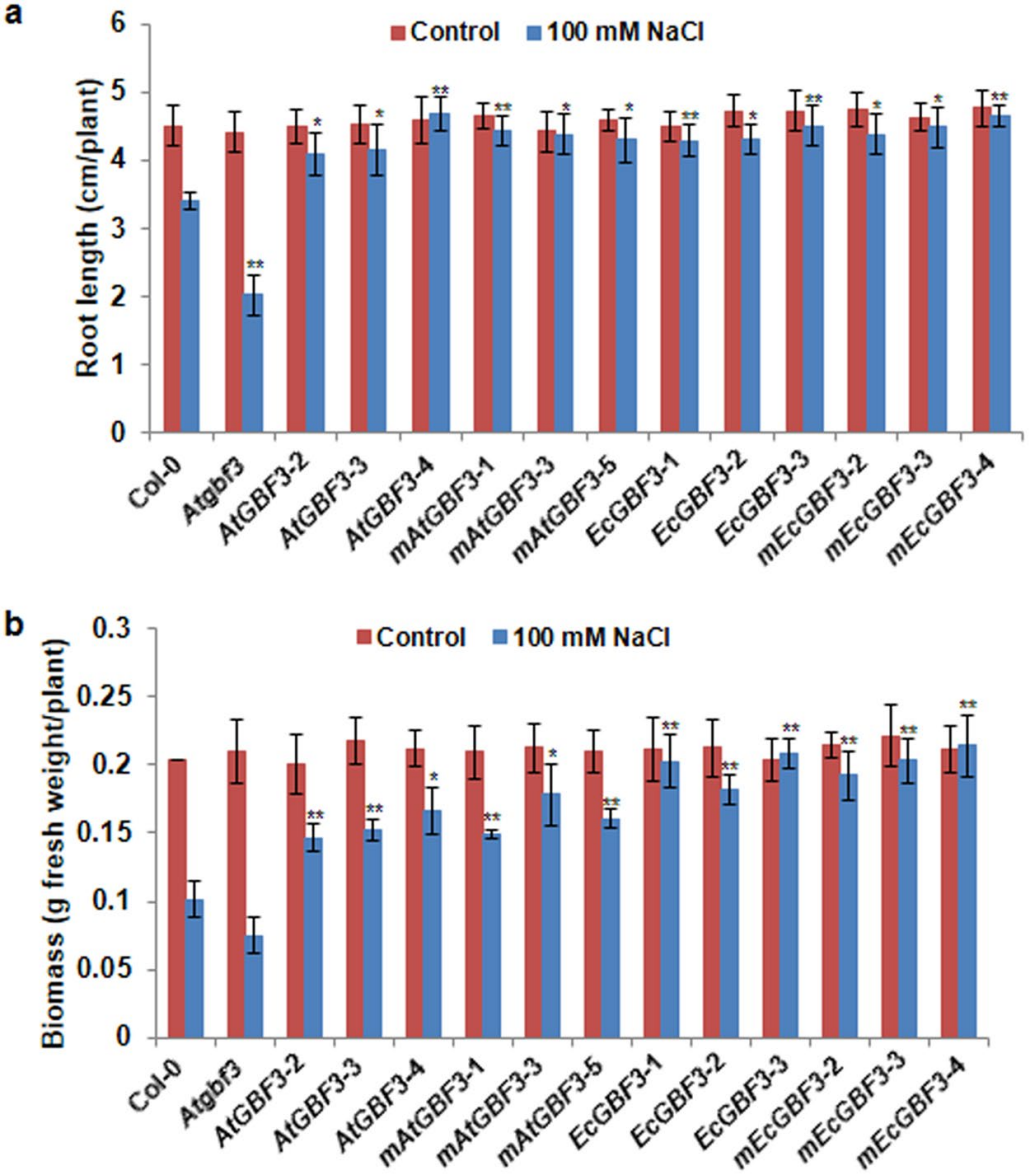
Improved salinity tolerance of *AtGBF3* and *EcGBF3* transgenic Arabidopsis plants. A week old hygromycin resistant T_3_ transgenic seedlings along with Col-0 and *Atgbf3* mutant seedlings were transferred on to medium (1/2 MS, 0.5% sucrose and 0.1% phytagel) containing 100 mM NaCl and growth performance was measured after 10 days. Root length (**a**) and biomass (**b**) were measured using plants carefully uprooted from the media and blotted on paper towel to remove any media. The values are means ± SE (n = 10). *t* test was used to show the statistical significance of the differences in the means of *Atgbf3* mutant or transgenic plants from Col-0 plants. * and ** indicate significant difference from Col-0 plants at *P* ≤ 0.05 and *P* ≤ 0.01, respectively. *Atgbf3*, *AtGBF3* mutant; *AtGBF3*-2, -3 and -4, transgenic lines overexpressing *AtGBF3*; *mAtGBF3*-1, 3 and -5, transgenic lines overexpressing *AtGBF3* in the *Atgbf3* mutant background; *EcGBF3*-1, 2 and 3, transgenic lines overexpressing *EcGBF3*; *mEcGBF3*-2, 3 and 4, transgenic lines overexpressing *EcGBF3* in the *Atgbf3* mutant background.

**Figure 5:**
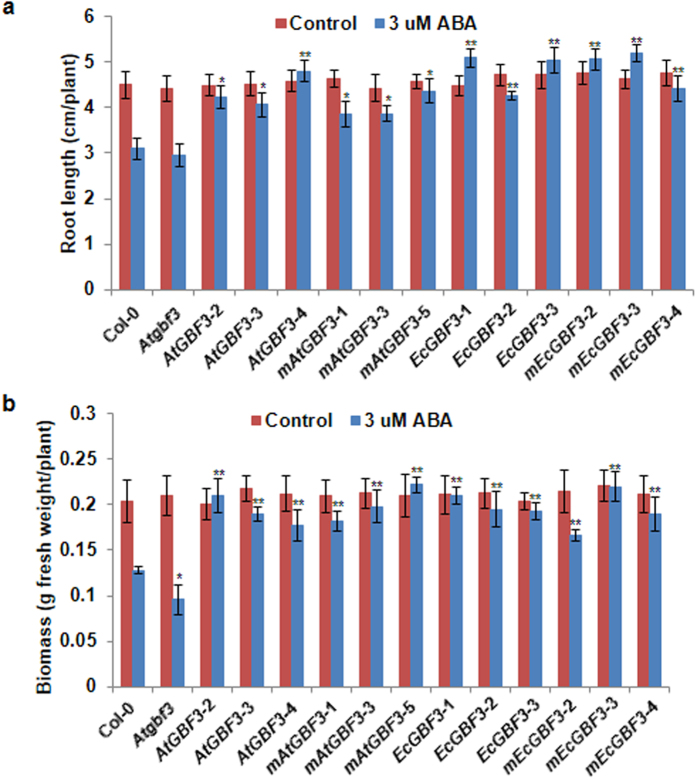
Insensitivity of *AtGBF3* and *EcGBF3* transgenic Arabidopsis plants to ABA. A week old hygromycin resistant T_3_ transgenic seedlings along with Col-0 and *Atgbf3* mutant seedlings were transferred on to medium (1/2 MS, 0.5% sucrose and 0.1% phytagel) containing 3 μM ABA and growth performance was measured after 10 days. Root length (**a**) and biomass (**b**) were measured using plants carefully uprooted from the media and blotted on paper towel to remove any media. The values are means ± SE (n = 10). *t* test was used to show the statistical significance of the differences in the means of *Atgbf3* mutant or transgenic plants from Col-0 plants. * and ** indicate significant difference from Col-0 plants at *P* ≤ 0.05 and *P* ≤ 0.01, respectively. *Atgbf3*, *AtGBF3* mutant; *AtGBF3*-2, -3 and -4, transgenic lines overexpressing *AtGBF3*; *mAtGBF3*-1, -3 and -5, transgenic lines overexpressing *AtGBF3* in the *Atgbf3* mutant background; *EcGBF3*-1, 2 and 3, transgenic lines overexpressing *EcGBF3*; *mEcGBF3*-2, 3 and 4, transgenic lines overexpressing *EcGBF3* in the *Atgbf3* mutant background.

Three lines from each *EcGBF3* and *AtGBF3* overexpressing plants both in WT and mutant background were selected for further stress response analysis. One week-old WT, *Atgbf3* mutant and transgenic seedlings either overexpressing *EcGBF3* or *AtGBF3* in the WT or mutant background were transferred onto MS medium supplemented with 100 mM mannitol, 100 mM NaCl or 3 μM ABA and growth was measured after 10 days (Supplementary Methods). Under non-stress condition no obvious phenotypic difference was observed between the WT and *Atgbf3* mutant (Supplementary Fig. S14a). Similarly, overexpression of *GBF3* in Arabidopsis also exhibited normal phenotype (Supplementary Fig. S14a). Under mannitol induced osmotic stress, all the plants showed narrow leaves (Supplementary Fig. S14b) with nearly 23% and 29% reduction in root length and biomass of WT plants (Fig. 3a and b). However, *Atgbf3* mutant plants showed 28% and 19% reduction in root length and biomass, respectively compared to WT plants (Fig. 3a and b). Transgenic plants expressing *GBF3* in the WT background showed improved tolerance to osmotic stress (Fig. 3a). More than 31% increase in root length was observed in transgenic plants compared to WT plants. Similarly, transgenic plants accumulated 34% higher biomass compared to WT plants (Fig. 3b). Expression of *GBF3* in the *Atgbf3* mutant background also improved the osmotic stress tolerance of mutant plants similar to *GBF3* overexpression plants. Results from the salinity stress experiment revealed sensitivity of *Atgbf3* mutant plants compared to WT plants (Fig. 4a; Supplementary Fig. S14c). The root length and biomass of mutant plants was 40% and 26% less compared to WT plants (Fig. 4a and b). Plants expressing *GBF3* in the WT or mutant background showed 22% and 44% increase in root length and biomass compared to WT plants, respectively. Similar observations were also made in plants treated with ABA wherein *Atgbf3* mutant showing greater sensitivity with about 6% and 25% reduction in root length and biomass compared to WT plants, respectively (Fig. 5a and b; Supplementary Fig. S14d). Plants expressing *GBF3* in WT or mutant background showed insensitivity to ABA with more than 22% and 33% increase in root length and biomass compared to WT plants, respectively (Fig. 5a and b). Taken together, these results indicate that loss-of-function of *AtGBF3* increases the sensitivity of Arabidopsis plants to osmotic stress, salinity and ABA treatment. Gain-of-function either by overexpression of *EcGBF3* or *AtGBF3* in both WT and mutant background increased the tolerance of Arabidopsis plants to osmotic stress and salinity while exhibiting insensitivity to ABA.

### Response of *GBF3* gain-of-function and loss-of-function Arabidopsis plants to water deficit stress

**Figure 6:**
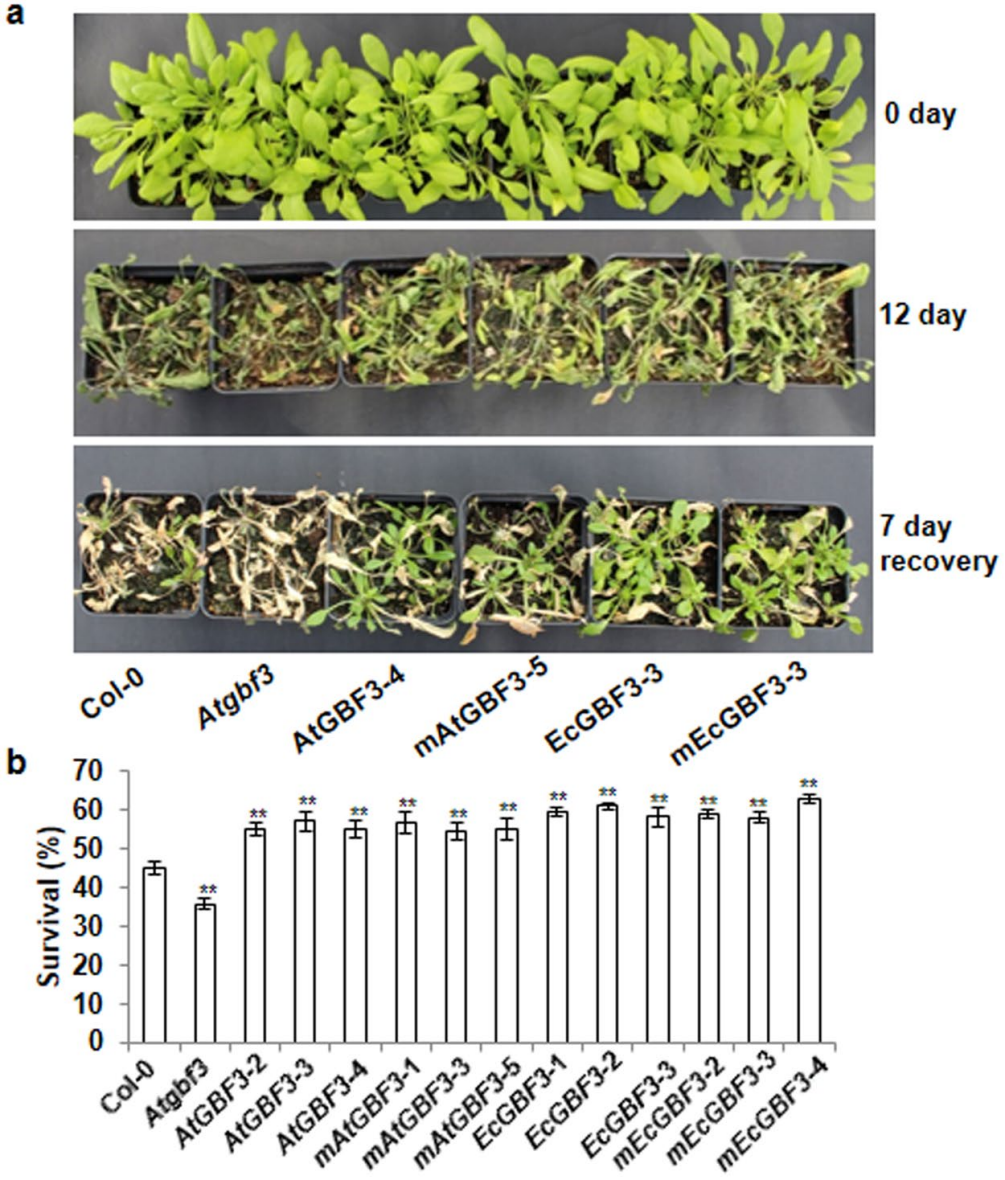
Enhanced drought tolerance of *AtGBF3* and *EcGBF3* transgenic plants. Drought stress was applied by stopping water on three week old plants for 12 days followed by re-watering for recovery. Phenotype of plants at 0 day drought, 12 days drought and a week after re-watering (**a**) and Seedling survival as determined by counting the number of plants with at least one newly grown leaf at the end of recovery period (**b**). The values are means ± SE (n = 10). *t* test was used to show the statistical significance of the differences in the means of *Atgbf3* mutant or transgenic plants from Col-0 plants. * and ** indicate significant difference from Col-0 plants at *P* ≤ 0.05 and *P* ≤ 0.01, respectively. *Atgbf3*, *AtGBF3* mutant; *AtGBF3*-2, -3 and -4, transgenic lines overexpressing *AtGBF3*; *mAtGBF3*-1, 3 and -5, transgenic lines overexpressing *AtGBF3* in the *Atgbf3* mutant background; *EcGBF3*-1, 2 and 3, transgenic lines overexpressing *EcGBF3*; *mEcGBF3*-2, 3 and 4, transgenic lines overexpressing *EcGBF3* in the *Atgbf3* mutant background.

Drought stress was applied to WT, *Atgbf3* and *GBF3* overexpressing plants. Under well-watered conditions no obvious phenotypic difference was observed among WT, *Atgbf3* mutant and *EcGBF3* or *AtGBF3* overexpression plants. Pot grown three-week old mutant and overexpression plants were subjected to drought stress by withholding irrigation for 12 days. *Atgbf3* mutant plants showed wilted and dried leaves whereas transgenic plants showed only mild wilting phenotype compared to WT plants (Fig. 6a). A week after re-watering, the number of survived seedlings with at least one fully expanded leaf was counted. *Atgbf3* mutants showed 20% reduction in survival compared to WT whereas *EcGBF3* or *AtGBF3* overexpressors showed more than 22% increase in survival compared to WT (Fig. 6b). *Atgbf3* mutant plants expressing *GBF3* also showed similar survival rates as overexpression lines. Together, these results indicate that loss-of-function of *GBF3* increases the sensitivity of Arabidopsis plants to drought stress whereas gain-of-function imparts drought tolerance.

### Potential biological function of GBF3

**Figure 7:**
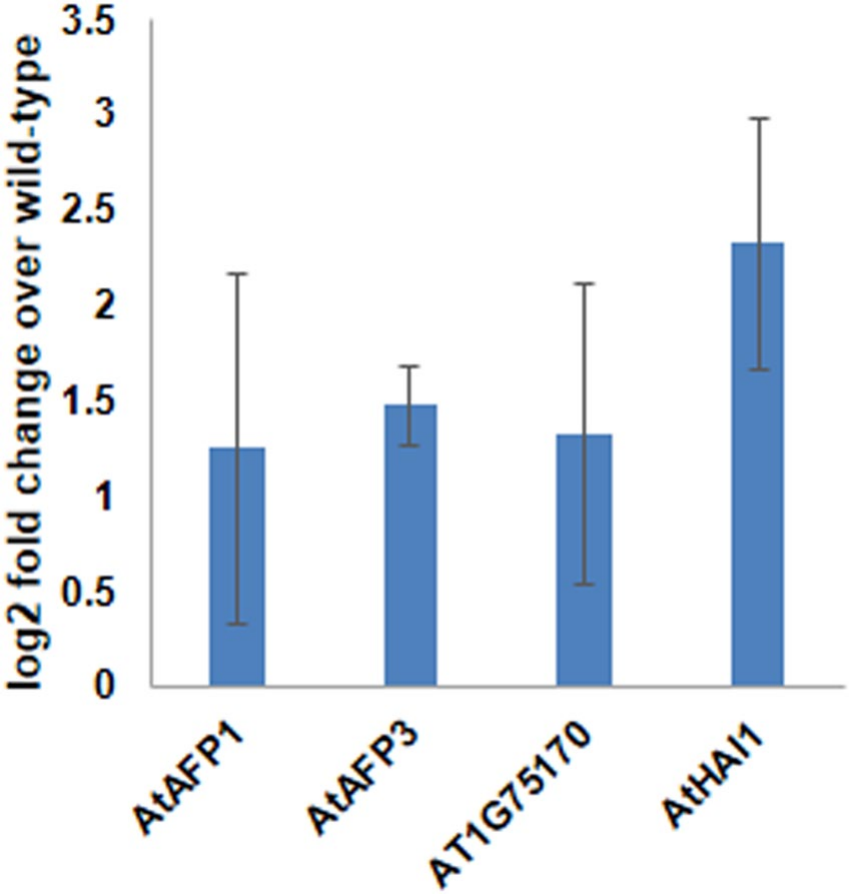
AtGBF3 overexpression increased the expression of ABA signaling genes. Expression of four potential targets of AtGBF3 was studied by RT-qPCR analysis in AtGBF3 overexpression plants. The data was normalized to *AtACTIN2* expression levels and the relative change over wild type plants was calculated using 2^−ΔΔCt^ method. The values are mean ± SE of three biological replicates.

A genome wide regulatory network which is based on expression data of 1400 transcription factors and their target genes was used to identify genes that are potentially regulated by AtGBF3. High confidence predicted targets which are positively regulated by *AtGBF3* with z score of ≥3.09 (q value ≥ 0.001) are given in the Supplementary Table S5. Further, to understand the biological processes that predicted targets of *AtGBF3* can regulate, biological process enrichment was performed using top 25 high confidence predicted targets. The GO terms with high significance levels converged into ABA mediated signaling pathway (Supplementary Fig. S16). Interestingly, six of the selected 25 genes for biological process enrichment were involved in ABA mediated signaling pathway (Supplementary Fig. S16). Based on the high confidence scores, the top five targets which also include three ABA mediated signaling pathway genes were selected to gain additional evidence. The consensus transcription factor binding sites (TFBS) on the promoter regions of these genes were predicted using The Plant Promoter Analysis Navigator (PlantPAN; http://PlantPAN2.itps.ncku.edu.tw). The regions selected for the TFBS prediction include 1 kb upstream of the transcription start site or until the preceding gene (whichever is minimum) and 5′ Untranslated Regions until the start codon. Four genes showed at least one G-box core motif (5′-CACGTG-3′) which is recognized by G-box transcription factors. The location of G-box core motif is given in Table 1. Expression analysis of *AtAFP1*, *AtAFP3*, AT1G75170 and *AtHAI1* genes in AtGBF3 overexpression plants showed increased expression of all the genes (Fig. 7). Further, the complete G-box element (5′-CCACGTGG-3′) on the promoter of alcohol dehydrogenase gene^31^ which was shown to be bound by GBF family transcription factors was found on the *AtAFP3* promoter (−137 to −130). Presence G-box motifs on the promoters and increased expression in AtGBF3 overexpression plants suggest that *AtGBF3*, *AtAFP1*, *AtAFP3*, AT1G75170 and *AtHAI1* genes work in a similar signaling pathway and AtGBF3 is the potential regulator of these genes.

**Table 1:** Potential targets of AtGBF3 with predicted G-box core motif on their promoters.

Sl. No	Locus ID	Description	Gene symbol	Position of G-box core (5′-CACGTG-3′)
1	AT1G69260	ABI five binding protein 1	AtAFP1	−536 to −531
2	AT3G29575	ABI five binding protein 3	AtAFP3	−92 to −87; −136 to −131; −174 to −169; −235 to −230; −290 to −285; −333 to −328
3	AT1G75170	Sec. 14p-like phosphatidylinositol transfer family protein	—	−174 to −169; −210 to −205
4	AT1G72770	Hypersensitive to ABA 1	AtHAB1	—
5	AT5G59220	Highly ABA-Induced PP2C gene 1	AtHAI1	−199 to −194

## Discussion

Photosynthesis is one of the major indicators of plant performance and is greatly affected by drought mainly through stomatal closure, membrane damage, and disturbed activity of various enzymes involved in ATP synthesis resulting in reduced biomass and yield^32^. The photosynthetic performance of finger millet was superior to that of rice, maize, *N. benthamiana* and Arabidopsis under severe drought stress suggesting its adaptation to arid and semi-arid regions at which drought is a common occurrence (Supplementary Fig. S1). We identified six genes from finger millet and validated their relevance in drought stress response. One such gene, *GBF3* is potentially involved in imparting drought tolerance as shown using loss-of-function and gain-of-function plants. Our experimental results on drought stress, ABA treatment and osmotic stress, explicitly confirmed the role of GBF3 in stress tolerance through potentially improved cellular protection in Arabidopsis.

The GBFs belong to G-group of bZIP superfamily transcription factors and have been shown to specifically bind to the G-box sequence in the promoter regions of several environmentally regulated genes^33, 34^. Till date, the plausible role of GBF3 has been implicated under light, cold, salt, osmotic stress and ABA treatment in Arabidopsis mainly by expression analysis^35, 36^. In our study, the expression of *GBF3* was highly induced under drought stress in finger millet, maize and Arabidopsis suggesting the conserved drought stress response of this gene across species. Overexpression of *EcGBF3* or *AtGBF3* resulted in improved tolerance of Arabidopsis plants to drought stress. Therefore, this study not only confirmed the relevance of GBF3 in drought tolerance but also provided confirmation that GBF3 orthologs in many plant species are expected to play this role. Furthermore, GBF3 overexpressing plants showed improved tolerance to salinity, osmotic stress and drought stress suggesting its role in multi-stress tolerance (Figs 3, 4, and 6). However, overexpression of GBF3 resulted in insensitivity of Arabidopsis plants to ABA (Fig. 5). As observed with GBF3 transgenic plants, several recent studies have shown improved tolerance of gene loss-of-function or gain-of-function plants to abiotic stress and insensitivity to ABA and *vice versa* ^29, 37, 38^. For example, overexpression of *OsPP108*, an ABA induced protein phosphatase 2C, increased tolerance of Arabidopsis plants to abiotic stress while imparting insensitivity to ABA^37^. Previously, a comprehensive meta-analysis for the effects of drought stress on photosynthesis and related molecular and metabolic events has identified four members of the bZIP (basic leucine zipper domain) family proteins that includes GBF3^39^. In addition, stress-induced expression of *GBF3* correlated with *Sucrose synthase 3* and *Neutral invertase* which are involved in grain filling^39^. Abiotic stress response of *AtAFP1*, *AtAFP3* and *AtHAI1* which are potentially regulated by *AtGBF3* has been shown through ABA signaling pathway (Supplementary Fig. S16)^40, 41^. The AFPs (ABI five binding proteins) are highly conserved plant-specific proteins that regulate ABA and stress response through their interactions with ABA-Insensitive 5 (ABI5) and related bZIP transcription factors. AFPs are shown to act upstream of ABI5^42^. Loss-of-function of *AFP* genes resulted in hypersensitive of Arabidopsis plants to ABA, whereas overexpression of *AFP* genes increased resistance^42^. GBF3 being a bZIP family transcription factor could potentially regulate the expression of AFP genes. Our results confirm that overexpression of AtGBF3 increased expression of *AFP* genes (Fig. 7) and increased resistance to ABA (Fig. S14d; Fig. 5). Therefore, our findings suggest a plausible role for GBF3 in improving drought tolerance in plants through regulation of *AFP* genes and increasing resistance to ABA.

Further, our study also provides resource for drought stress induced transcriptome as complete drought transcriptome data is not available so far in finger millet either as ESTs or by RNA-sequencing. In addition, the gravimetric method of drought stress imposition that we followed in this study simulates field situation resulting in coordinated expression of genes required for drought protection under field conditions. Therefore, the genes identified in this study are potential candidates for understanding the complex trait of ‘drought tolerance’ under field conditions. VIGS-based reverse genetics screen of finger miller orthologs in maize provide preliminary information on the plausible role of *RGAP2*, *DBH* and *RSLP* in regulating drought stress response. However, further characterization of these candidate genes through gain-of-function analysis will provide valuable information on their role in regulating and improving drought stress tolerance.

## Materials and Methods

### Plant growth conditions

Finger millet (*Eleusine coracana* cv. GPU28), maize (*Zea mays* ssp. *mays* cv. va35), rice (*Oryza sativa* ssp. *japonica* cv. Nipponbare) and *Nicotiana benthamiana* plants were grown in pots filled with equal weight of Redi-earth potting mix (SUNGRO Horticulture Distribution Inc., Bellevue, WA, USA) under greenhouse conditions at 28/22 ± 1 °C day/night temperatures, 600 μmol m^−2^ s^−1^ light intensity, 14 h/10 h day/night photoperiod cycles and 60–65% relative humidity (RH). Rice plants were maintained under flooded condition and other plants were maintained under normal water regimes until used for experiments. Nutrients were supplied to all plants every week using 24-8-16 Miracle-Gro (Scotts Miracle-Gro Products, Inc., Marysville, OH, USA).

Arabidopsis seeds were vernalized for 2 days at 4 °C and germinated in metro mix professional growing mix (SUNGRO, Horticulture Distribution, Inc., Bellevue, WA, USA) in a growth chamber (Conviron, Model CMP 4030, Winnipeg, Manitoba, Canada) set at 22/20 ± 1 °C day/night temperatures. The day/night cycles of photoperiod set were 8 h/16 h for first three weeks followed by 16 h/8 h. The light intensity was around 200 μmol m^−2^ s^−1^ and the RH was approximately 75%. Seeds of *Atgbf3* (SALK_082840 and SALK_067963) T-DNA insertional mutants were obtained from the Arabidopsis Biological Resource Center (Ohio State University; http://www.arabidopsis.org/). Genomic DNA was isolated using DNeasy plant mini kit (QIAGEN Inc. Valencia, CA, USA) and homozygous plants were confirmed by PCR analysis following the protocol described previously^43^. Primers were designed using SIGnAL iSect Tools (http://signal.salk.edu/isects.html) (Supplementary Table S1).

### Drought stress imposition protocol and assessing sensitivity of different crop and model plants

Initially, drought tolerance of finger millet, maize, rice, Arabidopsis and *N. benthamiana* was tested by exposing 25 days old plants to drought stress (35% FC) for five days. At the end of stress period, photosynthesis rate was measured. Finger millet plants showed relatively less reduction in photosynthesis rate under drought compared to rice, *N. benthamiana*, Arabidopsis and maize (Supplementary Fig. S1). This indicates that finger millet has relatively higher threshold for drought tolerance compared to other plant species studied here.

In order to construct subtractive cDNA library that includes early drought signaling genes, the following stress protocol was implemented. Drought stress was applied to 25 days old finger millet plants (Supplementary Fig. S2) and the reduction in soil and tissue water content were monitored. Soil water potential (Ψ_soil_) was reduced gradually from 100% FC (−0.04 MPa) to 35% FC (−1.1 MPa) (Supplementary Fig. S3a). Control and stress Ψ_soil_ were close to natural conditions in field *viz*, −0.03 MPa for well irrigated soil and −1.5 MPa for soil with less moisture where plants experience permanent wilting^44^. Leaf relative water content (RWC) decreased gradually with increase in stress severity (Supplementary Fig. S3b). The RWC was 92% at 100% FC and reduced to 50% at 35% FC. Similarly, membrane damage was gradually increased with increase in stress level (Supplementary Fig. S3c) and it was two-fold higher at 35% FC compared to 100% FC. The leaf osmolality also increased from 358 mmol kg^−1^ at 100% FC to 690 mmol kg^−1^ at 35% FC (Supplementary Fig. S3d) confirming the drought induced solute accumulation in the leaf tissue. These results indicate that the gravimetric method of drought stress imposition followed here was close to field situation and these stress levels are sufficient enough to capture both early and late drought stress responsive genes.

### Silencing vector construction

Fragments of finger millet orthologs of maize genes *ZmRGAP2* (GenBank accession number NM_001139255), *ZmDBH* (NM_001152987), *ZmGBF3* (BT063685), *ZmRSLP* (EU955694), *ZmUN* (NM_001148288) and *ZmHYP* (NM_001158082) were identified from public databases. All fragments were subject to siRNA SCAN tool (http://bioinfo2.noble.org/RNAiScan.htm; ref. 45) to identify candidate siRNAs, silencing efficiency and possible off targets (Supplementary Table S2). First strand cDNA derived from total RNA of 25 days old maize plants drought stressed until wilting was used as template to amplify gene fragments using specific primers flanked by *Avr*II and *Nco*I sites for restriction cloning (Supplementary Table S1). BMV-based VIGS vectors were obtained from Dr. Rick Nelson, Noble Research Institute LLC, USA. The gene fragments were cloned into *pBMV* vector in an antisense orientation^30, 46^. Fragment *ZmUBI7* (*Polyubiquitin7*; GenBank accession number NM_001153555) was cloned into *pBMV* vector to standardize VIGS and included in the experiment as a positive control. Inserts in all the constructs were confirmed by sequencing. The plasmids were mobilized into *Agrobacterium tumefaciens* strain GV2260 by electroporation.

### VIGS in maize plants

*N. benthamiana* plants were syringe inoculated with *A. tumefaciens* strain GV2260 carrying *pBMV* derivatives as described previously^47^. The inoculated plants were maintained under greenhouse conditions at 21 ± 2 °C for effective viral infection. A week after inoculation, sap was extracted from inoculated leaves by homogenizing in 0.1 M phosphate buffer (pH 6.0). The BMV titre of 10,000 relative expression units was used for maize inoculation. Six-day-old maize seedlings growing on pots filled with equal amount of uniformly mixed and completely dried potting mix were dusted with carborundum (Sigma-Aldrich, St. Louis, MO, USA) and leaf surface was gently rubbed with 50 μl of *N. benthamiana* sap. BMV-mediated systemic silencing in maize and the leaves used for VIGS experiments are described in Supplementary Fig. S6.

### Drought stress imposition on maize plants

Maize plants for VIGS experiments were grown on pots filled with equal weight of uniformly mixed and completely dried potting mix. At 14 days-post-inoculation (dpi) five each gene silenced plants were subjected to drought stress by withholding irrigation for four days. Soil moisture content was measured on volume basis using soil moisture meter (SM200 Soil Moisture Sensor, Delta-T Devices Ltd. Cambridge UK)^48^ (Supplementary Fig. S15a). Another set of five each gene silenced plants were maintained under well-watered condition as controls. As the potting mix used here was rich in organic matter content, organic soil calibration was used to convert the output from the sensor into per cent soil moisture content. Pots with nearly 20% (vol) moisture content were used for gene expression, physiological and biochemical analysis.

### Overexpression of *GBF3* in Arabidopsis

The complete coding regions of *AtGBF3* (At2g46270) and *EcGBF3* were amplified from cDNA derived from drought stressed leaf tissue, using specific primers (Supplementary Table S1) and cloned into *pMDC32* vector through Gateway recombination reaction (Invitrogen Corporation, Carlsbad, CA, USA). *pMDC32* vector alone with a fragment of *Green Fluorescent Protein* (*GFP*; 300 bp), *pMDC32::AtGBF3* and *pMDC32::EcGBF3* plasmids were mobilized into *A. tumefaciens* strain GV3101 by electroporation. Arabidopsis transgenic lines were generated by transforming ecotype Columbia-0 (Col-0) and *Atgbf3* mutant with *A. tumefaciens* carrying *pMDC32::AtGBF3*, *pMDC32:EcGBF3* or empty vector following floral dip method^49^. Integration of the construct was confirmed by PCR analysis of hygromycin (20 mg l^−1^) resistant T_1_ generation plants. Hygromycin resistant T_2_ plants from five lines per construct were used for transgene expression analysis by RT-qPCR using transgene specific primers (Supplementary Table S1). The data was normalized to *Elongation Factor 1α* expression levels and the relative change over vector transformed plants was calculated. Three lines per construct with high transgene expression were taken for further analysis.

### Drought stress imposition on Arabidopsis plants

Two inch plastic pots were filled with equal amount of uniformly mixed, autoclaved and completely dried potting mix. A week old hygromycin resistant T_3_ transgenic seedlings along with Col-0 and *Atgbf3* mutant seedlings were transferred onto pots. A week after the establishment, seedlings were thinned to ten equal sized seedlings per pot. Drought stress was applied by stopping water on three week old plants for 12 days. At the end of stress period soil moisture content on volume basis was measured using moisture meter (SM200 Soil Moisture Sensor, Delta-T Devices Ltd. Cambridge UK) (Supplementary Fig. S15b). Organic soil calibration was used to convert the output from the sensor into per cent soil moisture content. Pots with equal moisture content of nearly 10% (vol) were re-watered and seedling survival was determined after seven days of recovery.

### Soil water potential measurement

Soil water potential (Ψ_soil_) was determined using a WP4 dew-point potentiometer (Decagon Devices Inc., Washington, DC, USA) for soil samples collected from the root zone (a depth of 10 cm) of each stress levels under study according to the manufacturer’s protocol.

### Relative water content measurement

Leaf relative water content (RWC) was measured as described earlier^50^. Briefly, the leaf fragments of nearly same length and area were excised and fresh weight (FW) was measured immediately. Leaf fragments were hydrated to full turgidity by floating them on deionized water for 6 h, blotted on paper towel and the turgid weight (TW) was measured. Leaf fragments were then dried in hot air oven at 80 °C for 72 h and weighed to determine the dry weight (DW). The per cent RWC was calculated as

$${\rm{RWC}}( \% )=({\rm{FW}}-{\rm{DW}})/({\rm{TW}}-{\rm{DW}})\times 100.$$

### Cell membrane stability measurement

The leaf fragments (1 cm^2^) were harvested and rinsed in deionized water to remove the solutes leaked at the cut ends and then incubated in deionized water for 8 h at 25 °C under constant shaking (25 rpm). The conductivity of electrolytes leaked into bathing medium from stressed leaf samples was recorded (T1) using EC-TDS analyzer (CM183, Elico-India). Subsequently, leaf fragments were boiled for 30 min, and a final reading was recorded (T2) after cooling to room temperature. Similarly, conductivity was also measured for leaf segments from well-watered plants (C1 and C2). The cell membrane stability was calculated as described previously^51^ using the formula

$${\rm{Cell}}\,{\rm{Membrane}}\,{\rm{Stability}}\,(\mathrm{CMS} \% )=(1-\mathrm{T1}/\mathrm{T2})/(1-(\mathrm{C1}/\mathrm{C2}))\times 100$$

The loss of cell membrane stability under drought stress was expressed as per cent injury over well-watered plants. Injury was calculated as

$${\rm{Injury}}( \% )=(100-{\rm{CMS}})$$

### Osmolality measurement

Leaf osmolality was estimated using vapor pressure osmometer (VAPRO) (Wescor Inc., Logan, UT, USA) as described previously^52^. Briefly, leaf tissue collected from each stress levels was placed in 1.5 ml micro-centrifuge tubes with holes in the bottom and quickly frozen in liquid nitrogen. The tubes were allowed to thaw, placed inside another tube, and centrifuged at 6,000 rpm for 5 min. The osmolality of the eluted sap was measured.

### Photosynthesis measurements

VIGS plants were subjected to drought stress at 14 days-post-infiltration (dpi) by withholding irrigation for 5 days. Another set of plants was maintained at well-watered conditions as control. At the end of the stress period, photosynthesis rate and photochemical efficiency of PSII in a light-adapted state (Fv′/Fm′) were measured using second fully expanded leaf from top, using a portable photosynthesis system, LI-6400XT (LI-COR Inc., NE, USA) at CO_2_ concentration of 370 μ mol mol^−1^, light intensity of 1000 μ mol m^−2^ s^−1^ and RH of 55–60%. Instantaneous water use efficiency (WUEi) was calculated using net photosynthetic rate (A) and transpiration rate (T) as WUEi = (A/T).

### Genome wide regulatory network analysis of AtGBF3

The expression targets of AtGBF3 were predicted from a regulatory network of ~1400 Arabidopsis transcription factors and their predicted targets (Gupta and Pereira; unpublished data). Briefly, from a set of microarray expression datasets of Arabidopsis, genes that highly coexpressed with AtGBF3, with the partial correlation score significantly larger than the background, were marked as its targets. Using 25 high confidence predicted targets of AtGBF3 biological process enrichment analysis was performed (http://bioinfo.cau.edu.cn/agriGO/analysis.php).

## Electronic supplementary material

Supplementary figures 1-16 table 1-5 and methods
