# Supplementary material for: GBF3 transcription factor imparts drought tolerance in Arabidopsis thaliana
Source: Sci Rep. 2017 Aug 22;7:9148. doi: 10.1038/s41598-017-09542-1 (PMC5567290; doi:10.1038/s41598-017-09542-1)
Supplement: Supplementary file 1 — Supplementary figures 1-16 table 1-5 and methods [file 41598_2017_9542_MOESM1_ESM.pdf]

Supplementary figures 1-16
Supplementary tables 1-5
Supplementary methods

GBF3 transcription factor imparts drought tolerance in *Arabidopsis thaliana*

Venkategowda Ramegowda[1,2], Upinder Singh Gill[3], Palaiyur Nanjappan Sivalingam[3, 4], Aarti Gupta[5], Chirag Gupta[2], Geetha Govind[1], Karaba N Nataraja[1], Andy Pereira[2], Makarla Udayakumar[1], Kirankumar S Mysore[3, #] and Muthappa Senthil-Kumar[1, 3, 5, *]

[1]Department of Crop Physiology, University of Agricultural Sciences, Bengaluru, 560065, India; [2]Department of Crop, Soil and Environmental Sciences, University of Arkansas, Fayetteville, USA; [3]Plant Biology Division, The Samuel Roberts Noble Foundation, Inc. 2510 Sam Noble Parkway, Ardmore, OK 73401, USA; [4]ICAR-Central Institute for Arid Horticulture, Indian Council of Agricultural Research, Bikaner 334006, India; [5]National Institute of Plant Genome Research, Aruna Asaf Ali Marg, PO Box No. 10531, New Delhi 110 067, India
*corresponding author

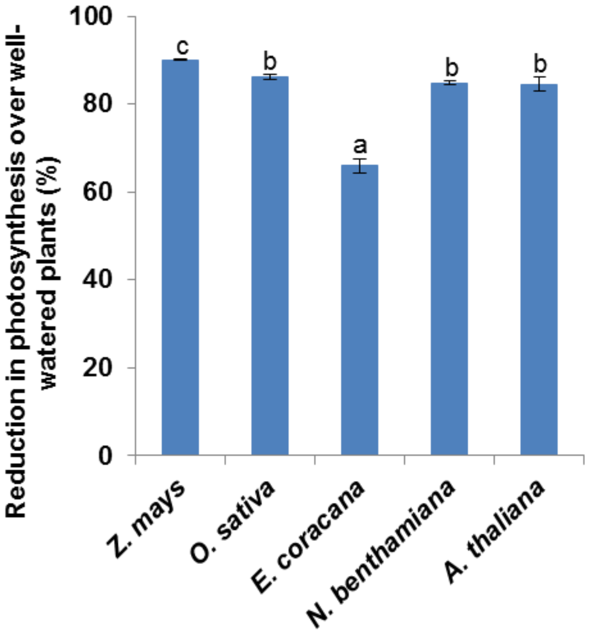

Fig. S1 Photosynthetic performance of different plants species under drought stress. Drought stress was imposed on 25-day old plants by following gravimetric method (Ramegowda et al. 2012). Plants were weighed daily at 10 am and maintained at 35% field capacity (FC) for five days by replenishing the water lost through evapotranspiration. A set of plants were also maintained at well-watered conditions (100% FC). At the end of stress period, photosynthesis rate was measured on second or third fully expanded leaves from top using a portable photosynthesis system, LI-6400XT (LI-COR Inc., NE, USA) at CO_2 concentration of 370 μmol mol^{-1}, light intensity of 1000 μmol m^{-2} s^{-1} and relative humidity of 55-60%. Per cent reduction in photosynthesis rate under drought stress over well-watered plants was calculated. The values are means ± SE (n=6); $P<0.01$, one-way ANOVA.

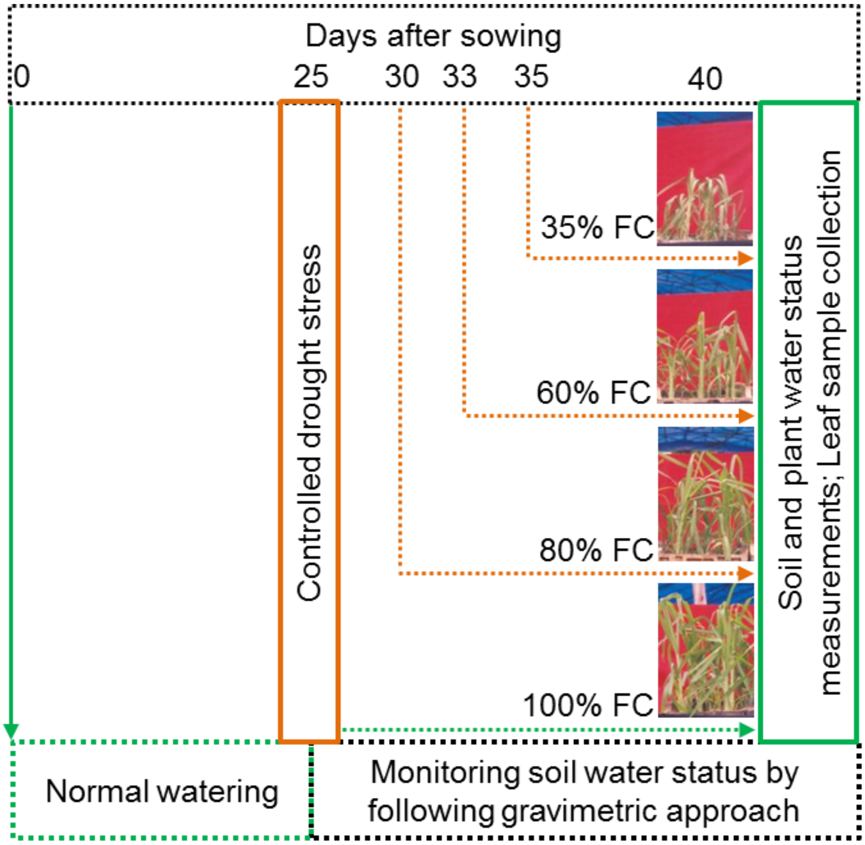

Fig. S2 Simplified representation of drought stress imposition in finger millet plants following gravimetric approach. Soil moisture status was gradually brought down to 80%, 60% and 35% field capacity (FC) by weighing the pots daily. Plants reaching respective FC's were maintained at that level by replenishing the lost water through evapotranspiration until the other set of plants reaching 35% FC and plants were allowed to acclimate for 5 more days. The inset photographs were taken at the end of stress period at respective FC's before the leaf sample collection for cDNA library construction.

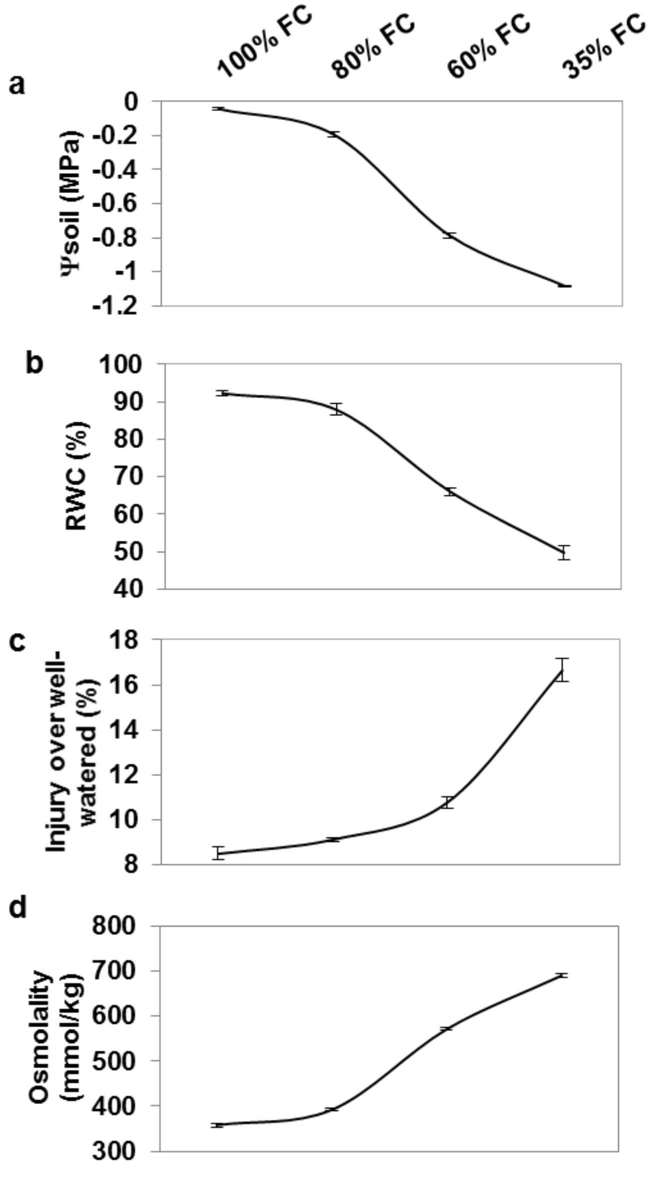

Fig. S3 Soil and tissue water status at different drought stress levels. Soil sample from the root zone and tissue from top second and third fully expanded leaves were collected at the end of stress period. Soil water potential (Ψ_{soil}) (a), Relative water content (b), Cell membrane stability (c) and Osmolality (d) were measured. The values are means ± SE (n=5).

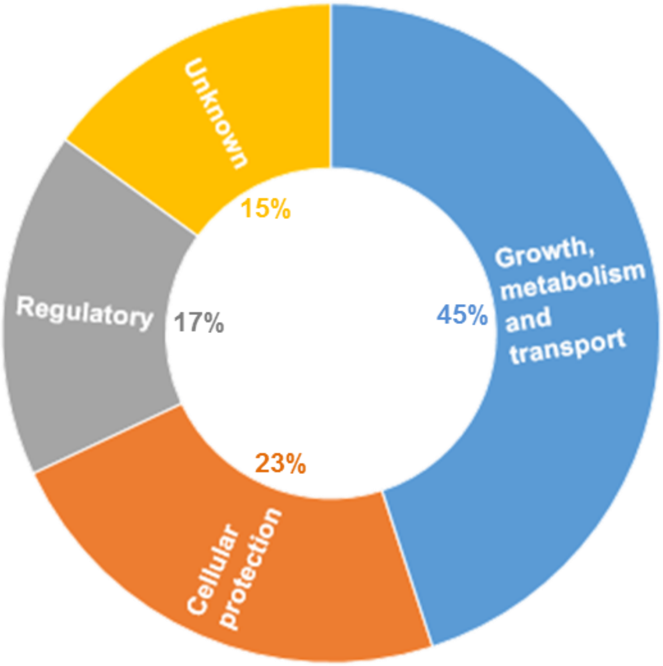

Fig. S4 Functional classification of ESTs from finger millet subtracted drought stress cDNA library. Homology searches were performed via BLASTx against the NCBI non-redundant (nr) database and putative functions were assigned based on the closest hits. Based on the putative functions ESTs are classified into different functional categories.

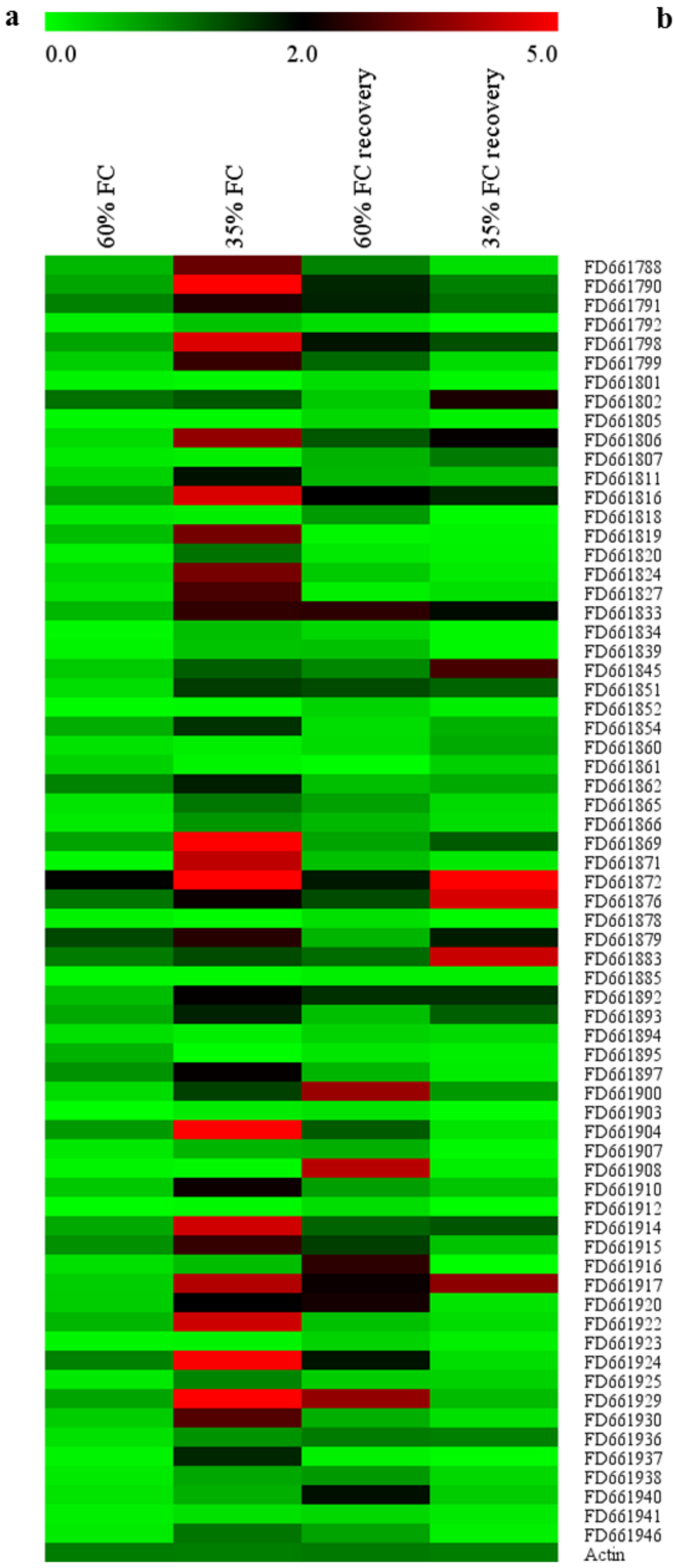

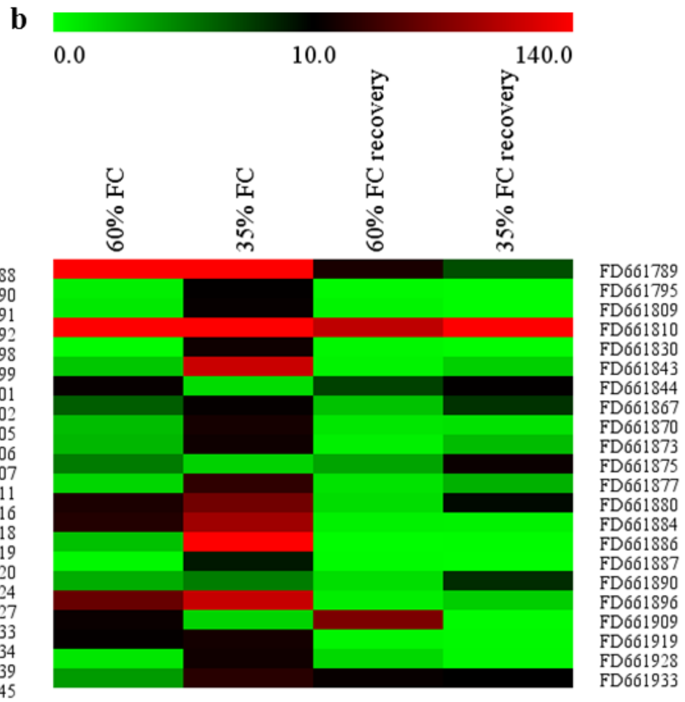

Fig. S5 Validation of drought stress response of EST's by expression analysis. Twenty-five days old plants grown under greenhouse conditions were exposed to drought stress by gravimetric approach. At the end of stress period leaf tissue was harvested from drought stressed and well-watered plants. Another set of drought stressed plants were re-watered and tissue was harvested at 6 h of recovery. Total RNA from leaf tissue exposed to different stress levels and recovery were used for RT-qPCR analysis. Expression values were normalized to *EcActin* expression levels and the relative change in transcript levels compared to well-watered condition was converted into heat maps using the TIGR Multiexperiment Viewer (http://www.tm4.org/mev.html). ESTs with 0 to 5-fold change (a) and ESTs with 0 to 140-fold change (b) represented as heat maps. The color bars on the top indicate fold change in expression levels under a given condition when compared to well-watered; green for lower expression and red for higher expression.

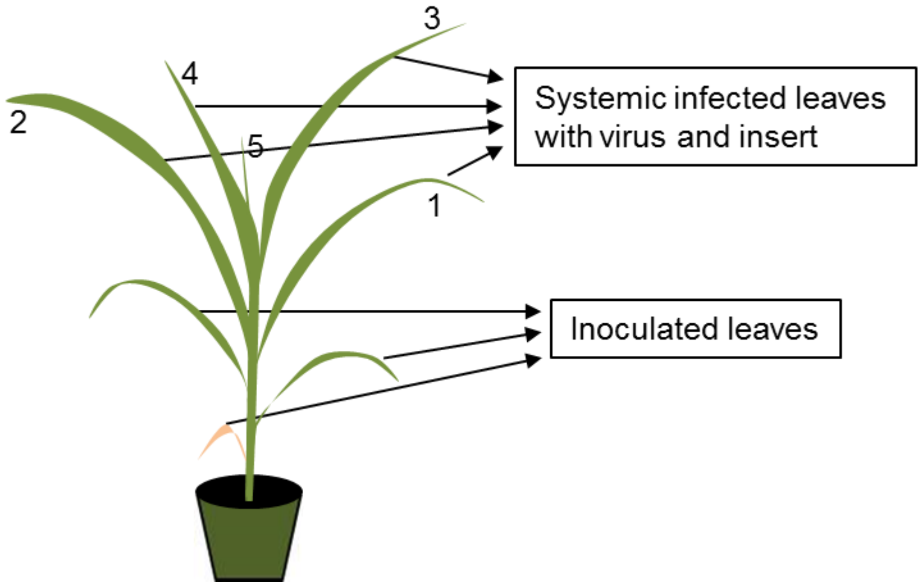

Fig. S6 Systemic virus-induced gene silencing (VIGS) in maize. BMV vectors carrying the fragments of gene of interest were inoculated on to the first, second and third leaves of six-day-old maize seedlings. At 11 dpi, systemic infection was found in 1-4 newly emerged leaves as analyzed by RT-qPCR for the presence of virus and insert. The third and fourth systemic infected leaves also showed silencing of gene of interest. Fifth leaf showed silencing >80% of the incidences and the degree of silencing was shown to vary as the systemic leaves advance (van der Linde et al. 2011). The third and fourth systemic infected leaves which showed gene silencing were used for physiological and biochemical analysis.

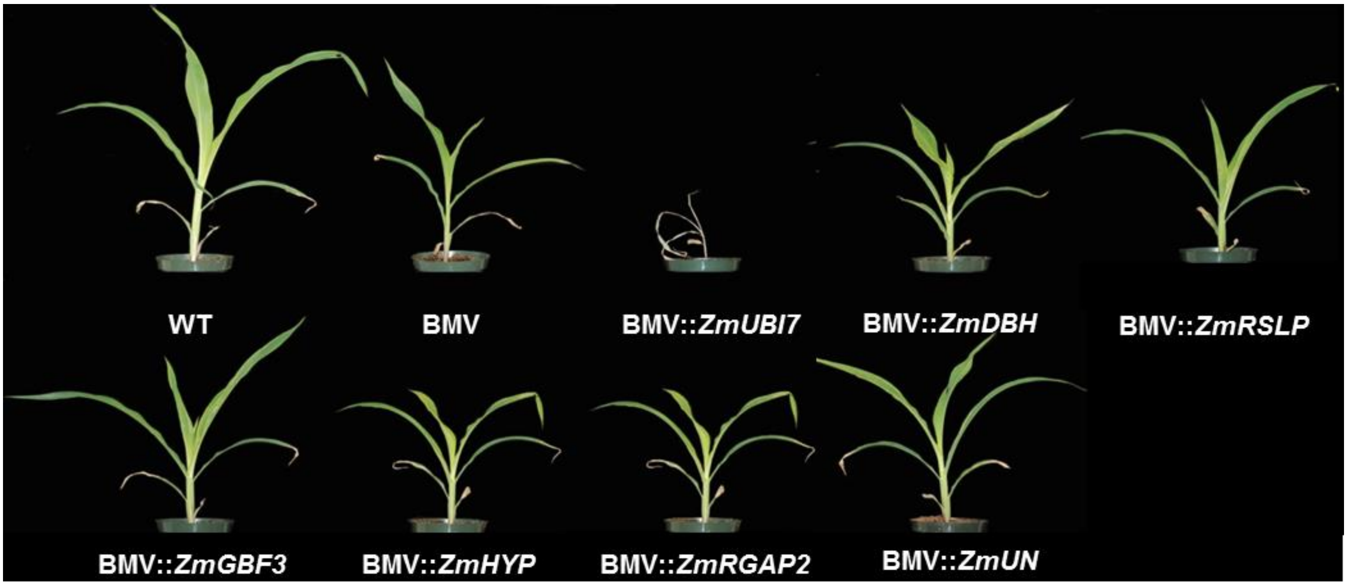

Fig. S7 Phenotypes of maize plants inoculated with BMV vectors carrying different maize genes. After inculcation plants were grown at normal conditions and photographs were taken at 14 dpi. BMV alone is known to cause only milder virus associated symptoms. In our experiments also some minor effects were observed in BMV vectors only inoculated plants. And hence interpretations on gene silenced plants in our experiments were in comparison to BMV vectors only inoculated plants. Silencing of housekeeping gene *ZmUBI7* resulted in lethal phonotype and hence it was not included in further analysis. WT, non-inoculated; BMV, vector inoculated; all others with BMV:: followed by gene name are respective gene-silenced plants.

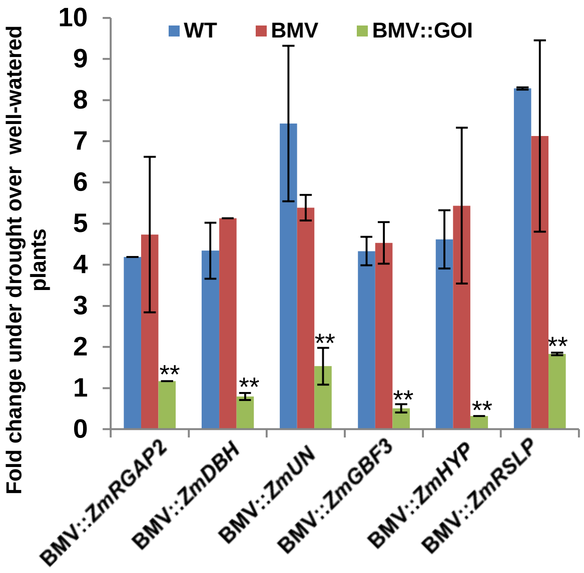

Fig. S8 Down-regulation of endogenous transcripts in gene silenced maize plants. Drought stress was applied at 14 dpi on all gene-silenced, BMV alone inoculated and WT non-inoculated plants by withholding irrigation for five days. Total RNA from leaf sample was used to analyze endogenous transcript levels of silenced genes in respective gene silenced plants by RT-qPCR. The expression values were normalized to *ZmActin* expression levels and the relative change over control was calculated using $2^{-\Delta\Delta Ct}$ method. The values are mean ± SE of three biological replicates. WT, non-inoculated; BMV, vector inoculated; all others with BMV:: followed by gene name are respective gene-silenced plants. ** indicates significant difference from BMV-inoculated plants at $P \leq 0.01$.

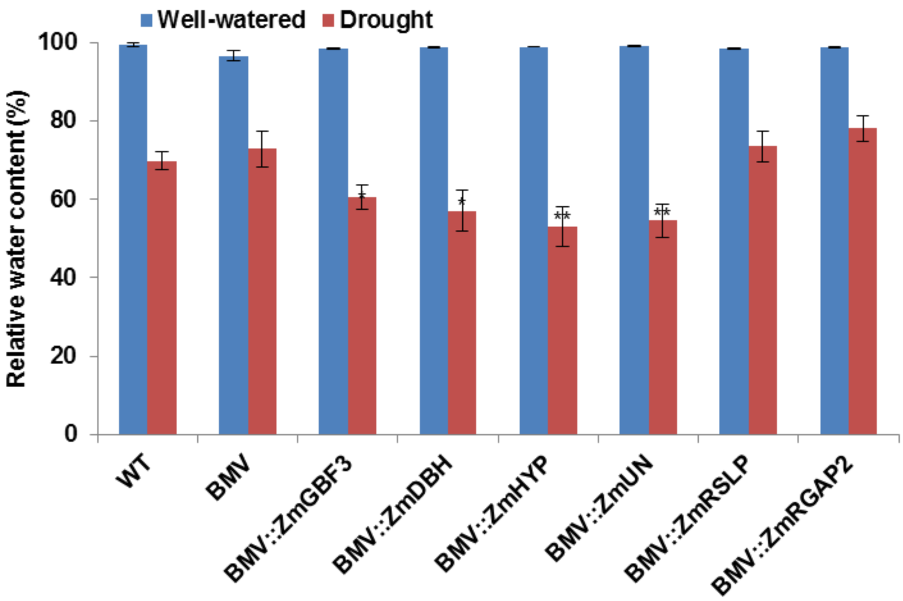

Fig. S9 Relative water content of silenced plants under drought stress. Drought stress was applied on plants at 14 dpi and relative water content was measured on fifth day. BMV-inoculated plants were used as reference for statistical analysis. The values are means ± SE (n=5). * and ** indicate significant differences from BMV-inoculated plants at $P \leq 0.05$ and $P \leq 0.01$, respectively. WT, non-inoculated; BMV, BMV vector inoculated; all others with BMV:: followed by gene name are respective gene silenced plants.

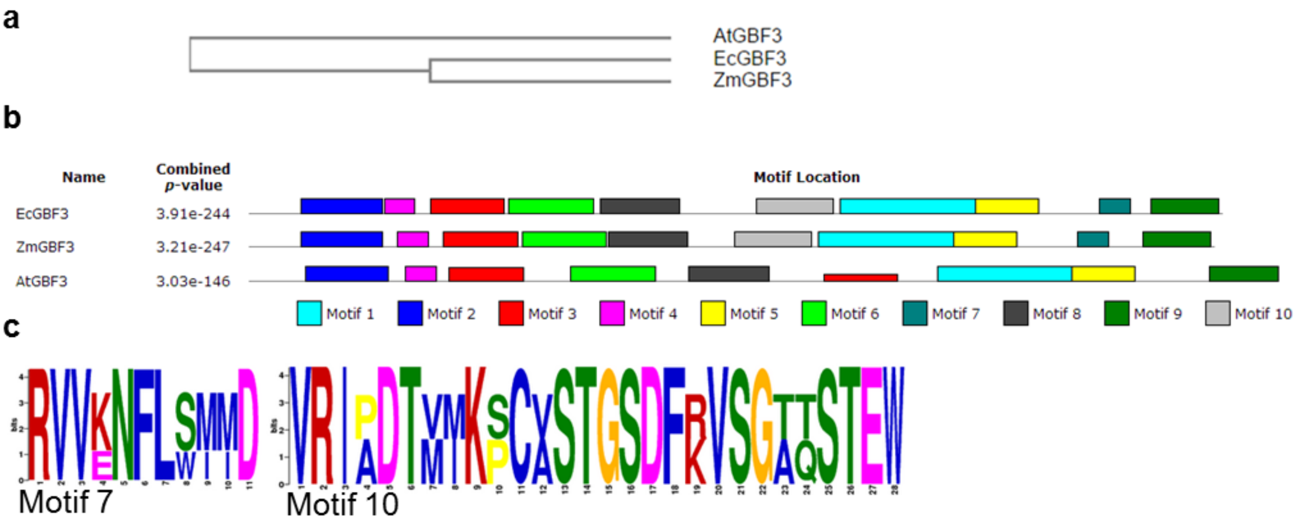

Fig. S10 Phylogenetic analysis and prediction of putative motifs in EcGBF3 and its orthologs. The deduced full-length amino acid sequences of EcGBF3, AtGBF3 (At2g46270) and ZmGBF3 (BT063685) were aligned using ClustalW and the phylogenetic tree was constructed using MEGA 4.0 by the Neighbor-Joining (NJ) method (a). Putative motifs were identified using MEME4.9.0 (http://meme.nbcr.net/meme/). Different putative motifs represented as numbered boxes (b). Motif 7 and Motif 10 present only in EcGBF3 and ZmGBF3 (c).

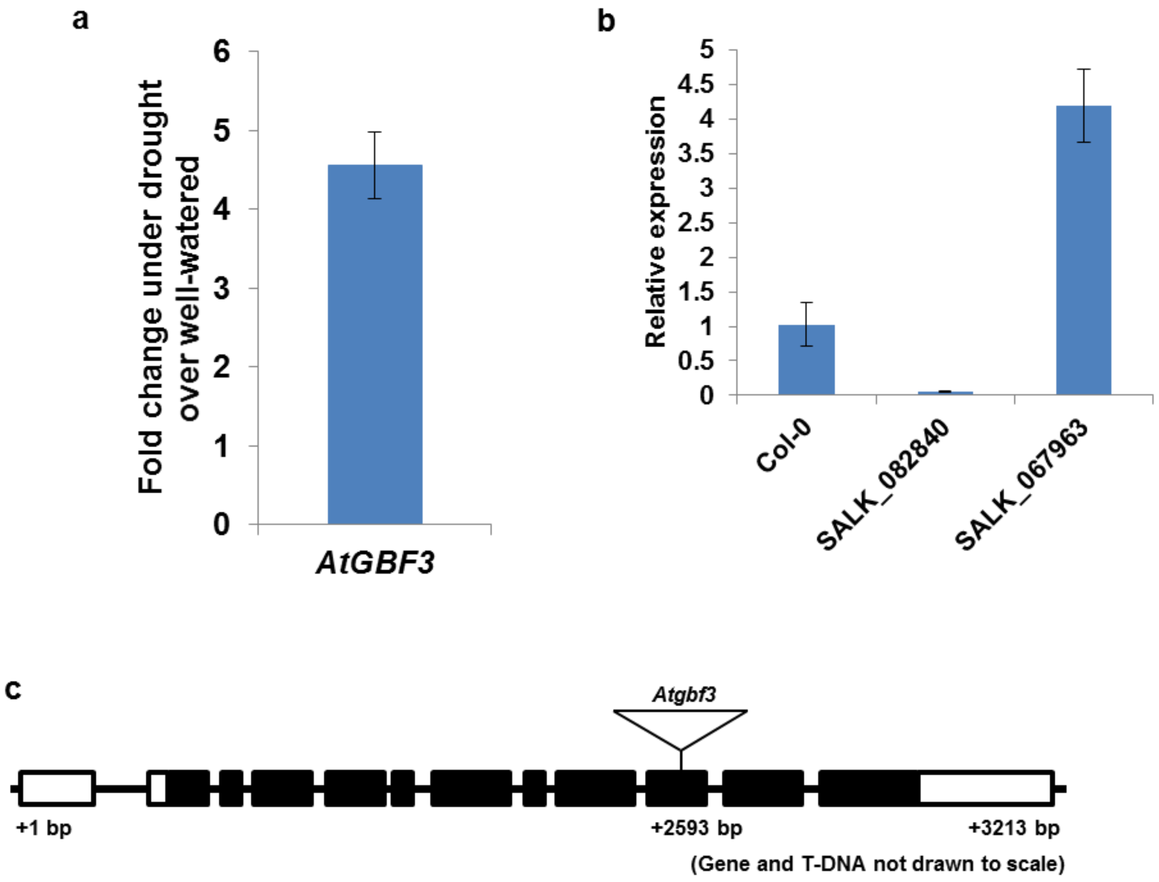

Fig. S11 Characterization of *AtGBF3* in Arabidopsis. Drought stress was imposed on 14 day old Arabidopsis plants until wilting. RNA from leaf tissue was used for expression analysis of RT-qPCR. Drought induced expression of *AtGBF3* in Arabidopsis (a) was measured. The data was normalized to expression levels of *Elongation factor 1*α (At5g60390). The relative change over well-watered plants was calculated using $2^{-\Delta\Delta Ct}$ method. The values are mean ± SE of three biological replicates. Relative expression levels of *AtGBF3* in wild-type (Col-0) and mutant plants (b). The relative change in expression was calculated over Col-0. Schematic representation of the T-DNA insertion in *Atgbf3* mutant (SALK_082840) (c).

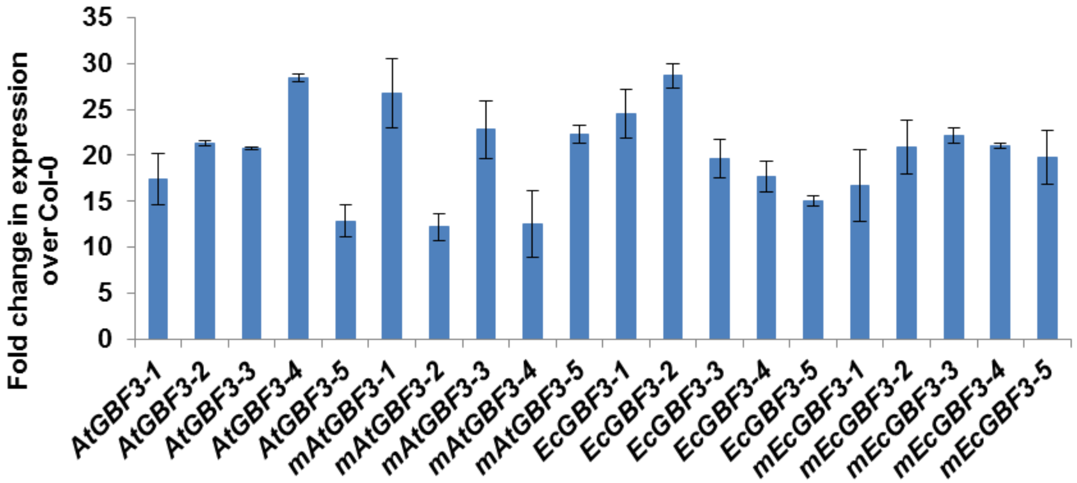

Fig. S12 Confirmation of transgene expression in Arabidopsis plants by RT-qPCR. Total RNA was isolated from leaves of hygromycin resistant T_2 plants grown under well-watered condition and used for RT-qPCR analysis with specific primers. Non-transformed Col-0 plants were used as controls for calculating the relative expression of *AtGBF3* and *EcGBF3* in transgenic plants. The data was normalized to *Elongation factor 1*α expression levels and the relative change over Col-0 was calculated. The values are mean ± SE (n=3). *AtGBF3*-1 to 5, transgenic lines overexpressing *AtGBF3*; *mAtGBF3*-1 to 5, transgenic lines overexpressing *AtGBF3* in the *Atgbf3* mutant background; *EcGBF3*-1 to 5, transgenic lines overexpressing *EcGBF3*; *mEcGBF3*-1 to 5, transgenic lines overexpressing *EcGBF3* in the *Atgbf3* mutant background.

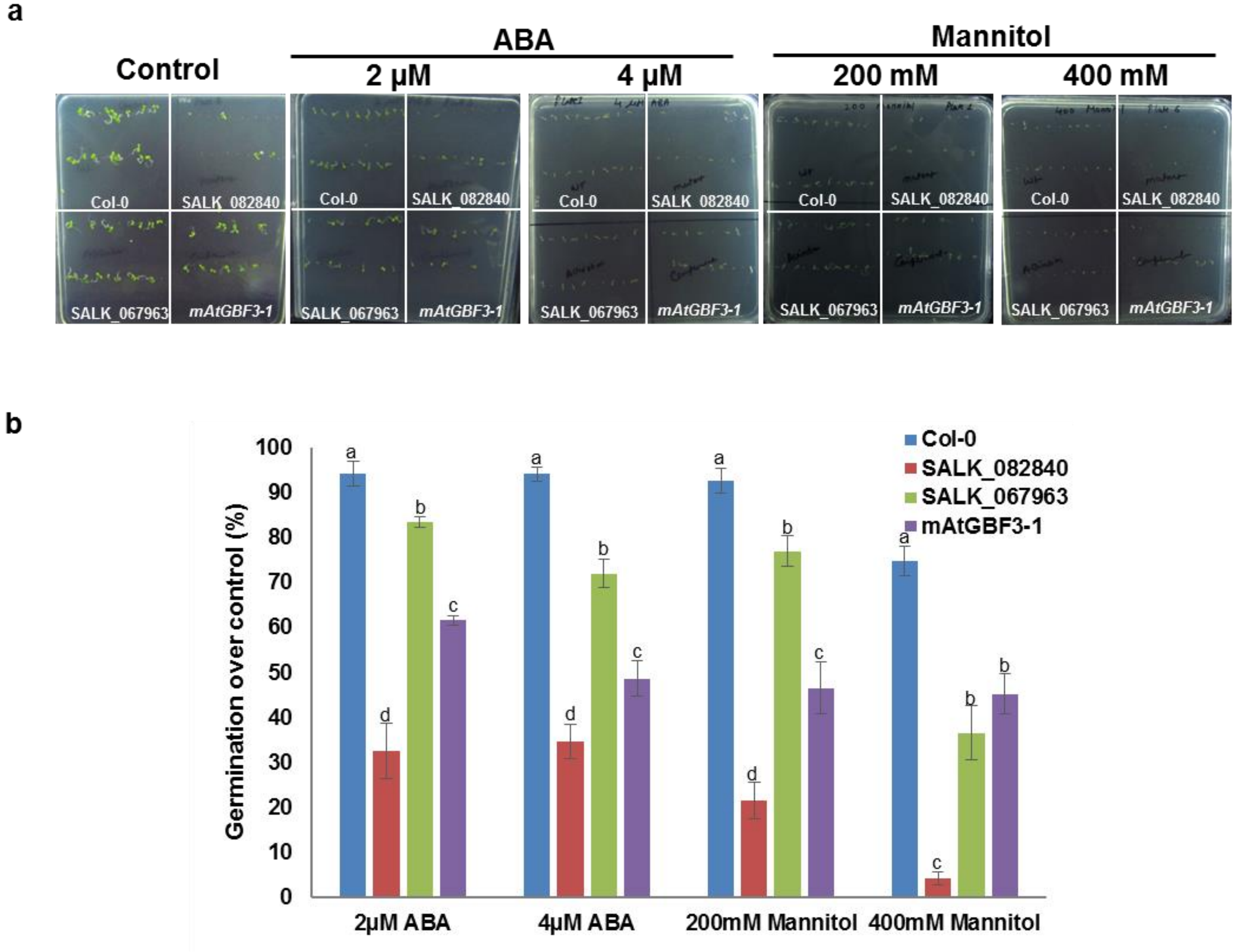

Fig. S13 Increased germination of *AtGBF3* activation and complementation lines under ABA and mannitol. Surface-sterilized and stratified seeds were sown on MS medium (0.8% agar and 3% sucrose) supplemented with different concentrations of ABA or mannitol. Germination rates were scored daily for seven days from sowing. Photographs were taken seven days after sowing (a). Percent germination in each treatment was calculated over their respective controls (b). The values are means ± SE of three independent experiments (n=20/line/experiment). [a, b, c, d] indicates that values are significantly different within the group (One-way ANOVA, generalized linear model procedure; SAS software). Col-0, wild type; SALK_067963, activation line of AtGBF3; SALK_082840, *Atgbf3* mutant; mAtGBF3-1, transgenic line overexpressing *AtGBF3* in the *Atgbf3* mutant background.

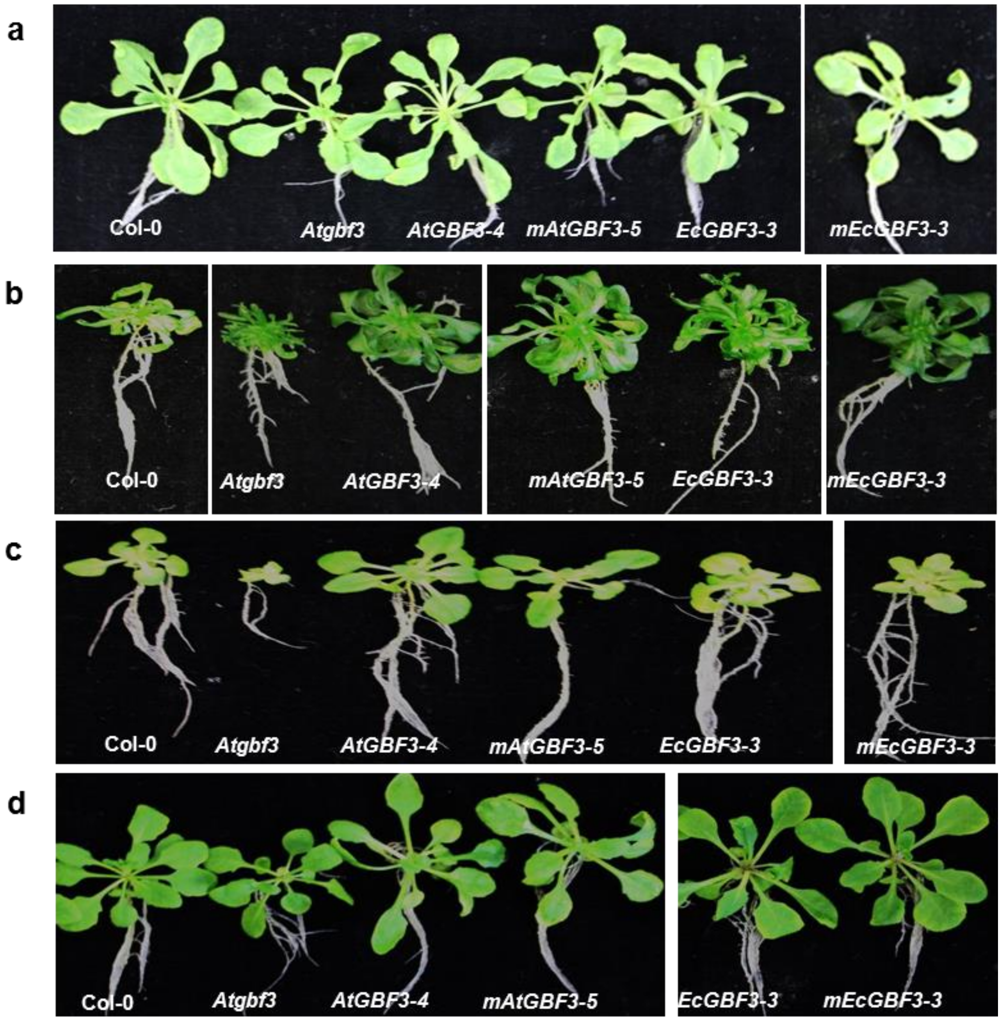

Fig. S14 Phenotype of Arabidopsis transgenics expressing *EcGBF3* and *AtGBF3* under different stresses. A week old T_2 generation hygromycin (20 mg l^{-1}) resistant seedlings were transferred on to medium (1/2 MS, 0.5% sucrose and 0.1 % phytagel) supplemented with different stressors. Photographs were taken from Control (a), 100 mM mannitol (b), 100 mM NaCl (c) and 3 μM ABA (d) treated plants carefully uprooted 10 days after stress treatment. *Atgbf3*, *AtGBF3* mutant; *AtGBF3*-4, transgenic line overexpressing *AtGBF3*; *mAtGBF3*-5, transgenic line overexpressing *AtGBF3* in the *Atgbf3* mutant background; *EcGBF3*-3, transgenic line overexpressing *EcGBF3*; *mEcGBF3*-3, transgenic line overexpressing *EcGBF3* in the *Atgbf3* mutant background.

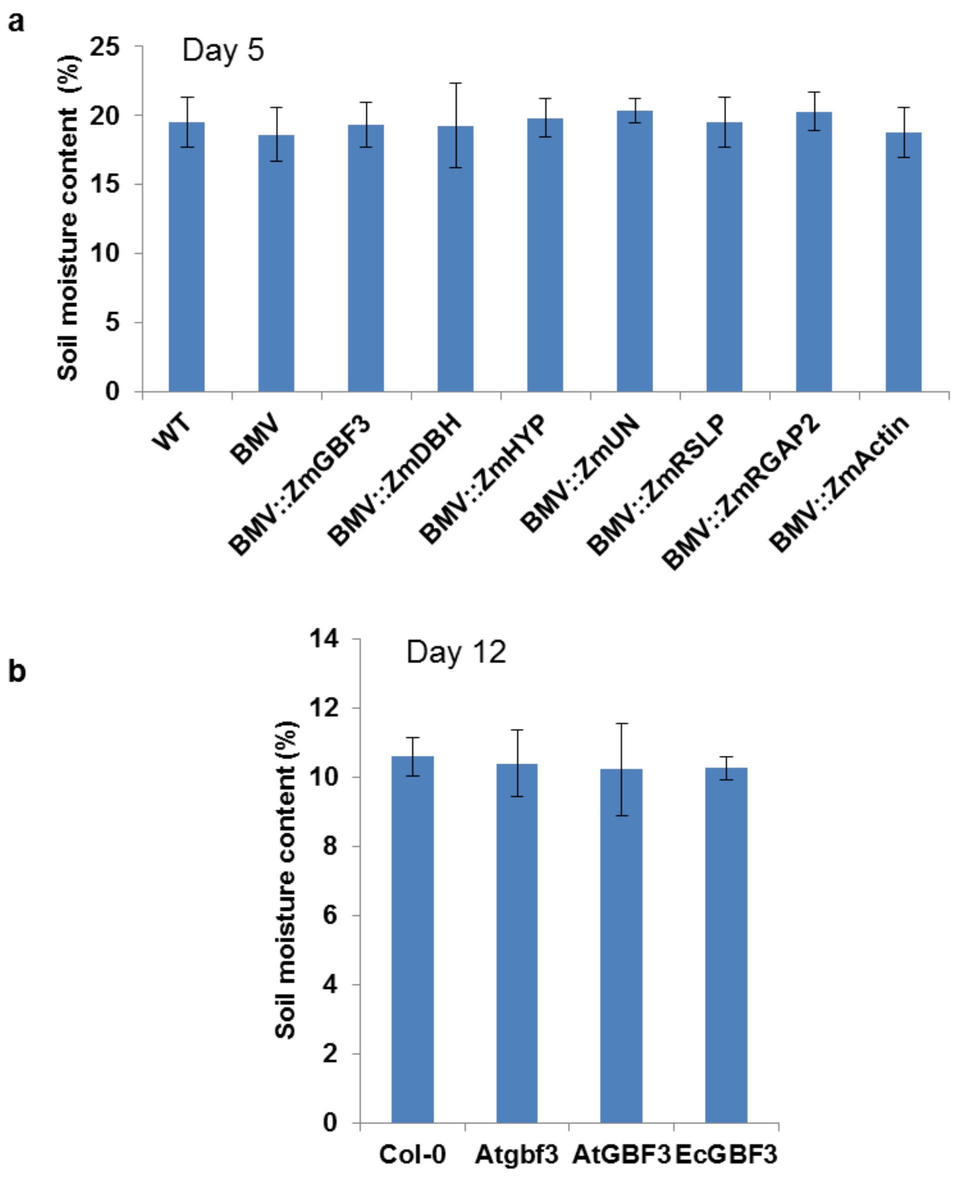

Fig. S15 Soil moisture content of plants subjected to drought stress. During VIGS experiment, maize plants at 14 dpi were subjected to drought stress by withholding irrigation for four days. Soil moisture content (a) was measured on volume basis using soil moisture meter at the end of stress period. The values are means ± SE (n=5). Drought phenotype of gain-of-function and loss-of-function of GBF3 in Arabidopsis was studied by subjecting three week old plants to drought stress by withholding irrigation for 12 days. Soil moisture content (b) was measured on volume basis using soil moisture meter at the end of stress period. The values are means ± SE (n=10). Soil moisture meter is supplied with two general soil calibrations to convert the output from the sensor into per cent soil moisture content. One for mineral soils (predominantly sand, silt and clay) and another for organic soils (with a high organic matter content). As the potting mix used here was rich in organic matter content, organic soil calibration was used to convert the output from instrument to per cent soil moisture content.

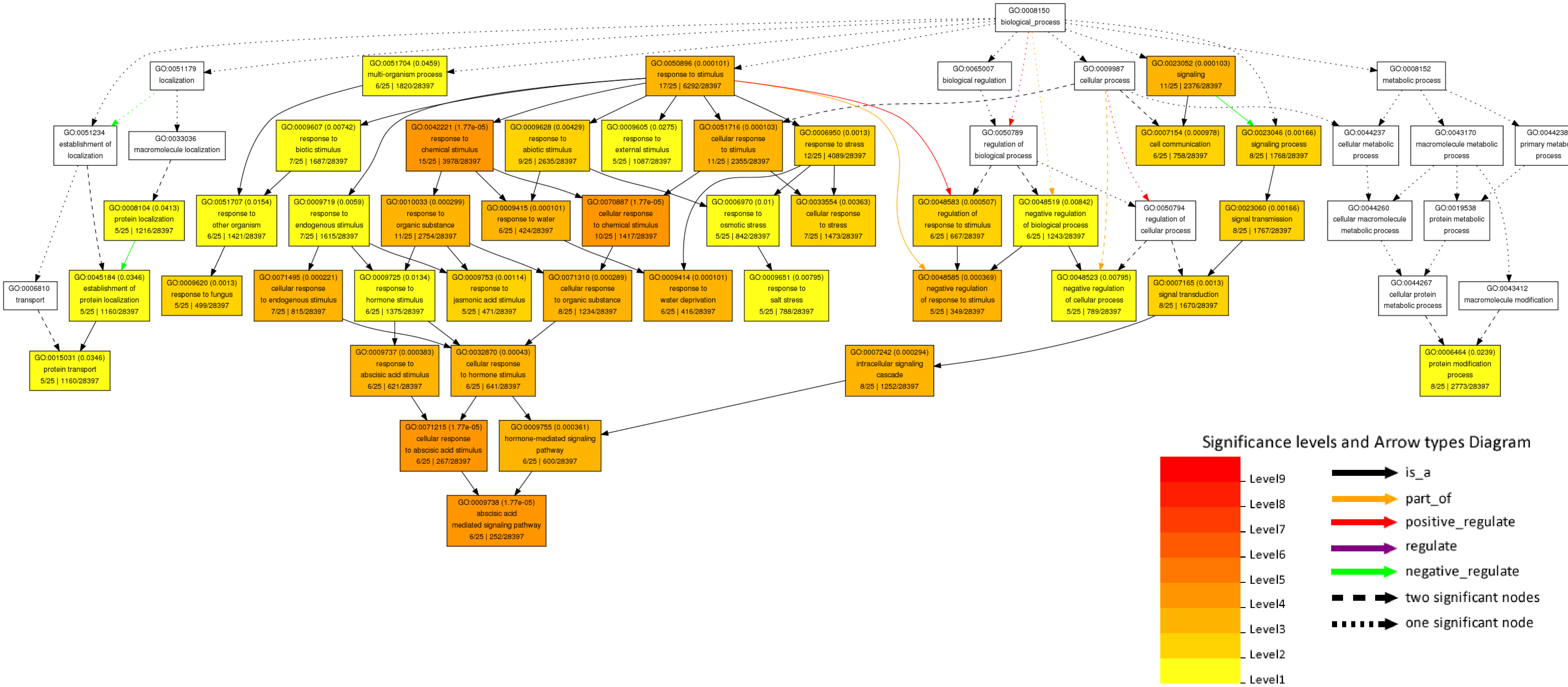

Fig. S16 Biological process enrichment of high confidence predicted targets of AtGBF3. Singular Enrichment Analysis (SEA) of AGRIGO (http://bioinfo.cau.edu.cn/agriGO/analysis.php) was used to perform biological process enrichment with 25 predicted targets of AtGBF3 with q value ≥0.001.

Table S1. Primers used in the study.

Primers used in qPCR analysis of finger millet ESTs

NCBI GenBank acc#	Primer (5′ to 3′)	Product size
FD661788	AACAGGCGCCAAAGTGGAATTCAG	208
	TAACATTTGCACCTGAGTCACCGC	
FD661789	TGACGTGGTTCGCGTTCAC	251
	AGCCCACTACAAGCTATACAGGCA	
FD661790	TGGCTGTTCAATTACGGTATCGGC	248
	TGCAGATCTCGATCATGAAGCCG	
FD661791	ATGAGCTGAGGGAGGAGAAGCAAA	228
	TATCCACATCTGAAGGCGGCATGA	
FD661792	TACGGCGAACAATCCCTACGTGAA	202
	GTAGACGAGCAATGCACGCAACAA	
FD661795	TTTGCTGCTGCTGCTTGAGGGAAA	204
	GGCTCAAAGAAGTGGGCATCC	
FD661798	AAGATTGACGAGAATGTGGCGAGG	236
	TGCATGAAGTGGCAGTAACTCACG	
FD661799	ATGCCCAGAAGGATCAAGAACGGA	229
	ATCTTTGAGACGGCCTTGTTTGCG	
FD661801	GCGCTCACAAGACAAGTCACTGAA	225
	AACTCACCGGTAAGGTATGACGGG	
FD661802	ACGCGGCAATGAAGAAAGTACTGG	292
	TACAGGCAGCAGCAACGGTAGTTA	
FD661805	GCCGAAGGTATCTAAGGTCCATCA	215
	CCCAGCAGGGCAATTAACAGCTTA	
FD661806	AGAACCAAGGGTGGTTCCTGATC	247
	AAGACAGCCGATGTGTGGTCTTG	

FD661807	AAGGATGGGAGAGAAGAAGCCTG<br>TGCACGTACCATCGACTATTGCAG	200
FD661809	AGAAGGGTGCAGGCTGGTG<br>AGCAGCGGGTGATCAAAGTAGAGA	244
FD661810	AGGGCATCATGGACAAGATCAAGG<br>AGACTCTTCACACTGCCCACAACA	204
FD661811	TACATCGGCTACTCCAACATCTGC<br>TTCCAGGCACAACAGCTCGCAAA	247
FD661816	TGTGGCGAGGGAGATCATAAACCA<br>TGCATGAAGTGGCAGTAACTCACG	222
FD661818	AGCAATGGTGGCCTCTCATGATC<br>TACTTGTCGACGAACTGGTACACG	236
FD661819	TTCATCGACCTCCAGCTATGACGA<br>ATGATGGTTCCAGCAACACCAACG	200
FD661820	AGTGAGAGAGCGAGAGATACGCTG<br>TCTCACTCTGCTACGCTGACTAC	282
FD661824	GATGTGTTCGCAAAGCCGATTCCA<br>GTCACGATTTCACAGGCCATTCCA	210
FD661827	AATCTTCACCAAGCCAGTGGGAGA<br>AGCTAGCTTGGCTTCAAAGGGAC	205
FD661830	TTCCGGGAACTCCTTAATCTGCTC<br>CGCAGCCAGTATAAACAGACGGTA	241
FD661833	AAGATATGGTGGTGGCAAAGCAGC<br>TTCAATCTTCTCACCCACGGTCCA	211
FD661834	AGGTGTACAACGTGCTGAGCAAAG<br>ATGGCCTGGTCGCTGAGTTTCATA	202
FD661839	CCC CGG GCC AAA GGC AGG C<br>CCC CCC GGG GAA GCC CCC	240

FD661843	AGAAGGAGGACAAGAACGACACC<br>CGTCGAAGCCTTAACCAGTGATCTGA	204
FD661844	AATCTGATGGCCGATGTTGTGGAG<br>AATGCCTTAACTGCTCCCAGTTGC	209
FD661845	AACAAGATGCTTTCATGTGCCGGG<br>TCAGTGCGGTTGAACTTGGTGAAG	245
FD661851	GACTACATTCCAAAGGCGCACACA<br>GCCTCCGCAATGCACGATATTCA	241
FD661852	AGAGTTTCCAAGGCAATCAGCGAG<br>AGCTCCTGTCTTGATTTGACCCG	213
FD661854	TGTCACGTGTCGCAAGACAAATGG<br>ATGGAGGATGTCGATGTGTGTGG	230
FD661860	TGATGAGATAGCAGAGCACGCTGA<br>CACCATGTTCACCACAAATGCCGA	249
FD661861	GCATCTTCATTCATGCCCTCA<br>TGCTGCCCGTTCAGTAACTCATC	200
FD661862	AACTTGTTCTGTGCACTTCACCGC<br>AGGCATGGGTAAAGCAGCGATTTG	206
FD661865	ATGACATGCGTGTGAGCACAACAG<br>ATCACTCCGTATCCCGTCGTTACA	228
FD661866	TCACAACCTCAGTCACGGACAACA<br>CGAGTTCTGCATATGCTCCGTCG	248
FD661867	TACCCACATCGTTCCTGTTGCTGA<br>TTTGAGGGCTCCCTGTCATGCTTA	231
FD661869	AGGTGCTCACCAACGGAAAGTACA<br>TCAAGCTTGCTAGAGAAGACGAGC	219
FD661870	TTCCTAATTGGCGGCAAGATGCAG<br>AACCCATCGTAGAAGCCACCGAA	205

FD661871	ATCACTACAACCAGCATCCTGTGC<br>TTCCAGAATACACACACAAGGCCC	201
FD661872	TGCCCGAAGAAGGACAACAGCTA<br>ATTTAGCCACATCCATTGGCAGCG	223
FD661873	ACTGAATCGACATCCTCATCGCCA<br>ACATTACCACCACAGGCAGTCGAA	204
FD661875	TGTGGACTGCAGTGGAGATGACAA<br>TTTGATATTCGTGATGCGCTGGGC	247
FD661876	TGAACGTGAGGAGCGTGCTAAGAA<br>TGTCGGGAATTCCGATTTGTCCTG	202
FD661877	AAGAGTTCATGGCCGGAGTTAGTG<br>CAAGCTTTGATCAAGCGAGCTGG	206
FD661878	CGTGATCGAGTTCATCCTCTTCCA<br>AGTTGTGCTGGATCAGGAATCCCA	246
FD661879	AGCAGCTAGGAGCAAGTTTCAAGG<br>ACAGTTGACTTGGAGGAACCACC	232
FD661880	TTTCGCATGTCGGTACATTGGTGG<br>TGCATGTGGGAGCTCAATTGCTTC	235
FD661883	AGGAGTTTGAGGAGTACGCTGAGA<br>GCTTCACCTTGGTGATGTAATCGG	228
FD661884	TGGCTCATGATGAAGCAGTGGCTA<br>AATATGTTGGTGGCAACACGCCAG	221
FD661885	TGCAGAACACCTGCCTCAAGA<br>AAGAGAAGAGTACGTAGTGACCGC	214
FD661886	GCCTTTGGCGATAAGAAGGAAGGA<br>ACACTGTTGTGTTACAACGCCAGC	204
FD661887	ATCAAGTACGGCGACGCCTTCAA<br>ACCGGGTCCTGGGTGATGTTGA	257

FD661890	AATGAATCCGGAGGATGAGGCTGA<br>TTCATCCACCTCCATTGCCTTGAC	249
FD661892	TGAACGTGAGGAGCGTGCTAAGAA<br>AGTGTCGGGAATTCCGATTTGTCC	202
FD661893	TGCCTTGTGCCCTATCCTTGCTA<br>AAACCCATGCCGTGTTCTTCTTCC	261
FD661894	ACGTTCAAGGTACACGCACACAAC<br>GGCTGATAAACCGCATTGATCCAC	221
FD661895	CTACACACCATAGACAACAACCGC<br>TCGAGGTCGAAGTAGACGCTCTTG	212
FD661896	AAGGAAATTGATGGGACTGCCGTG<br>TGCTAATCGGCCTGGTTCTCTTG	206
FD661897	ATCGACTGCTCCAGGGTCAGCTAAA<br>TCTTCTACGGAGCGTATAGGAGTG	206
FD661900	CTATGCAGCTGCTTTCCACCAAGA<br>TATTTCTTGCCACCACGTTCGCCA	200
FD661903	AAGGCAACGGGTTGAATTTCCAGG<br>TTCGAGCCTTGTCCATGAGTACGA	249
FD661904	TTTGCTGAGGCAAATGCTGAGGAC<br>TGATCCACCCTTGGAGCTGAATGA	232
FD661907	TGACAAGGTTAAAGGTGCCGATGG<br>CGTTCACCGAACGCATTATGAGCA	205
FD661908	GGAAGGTGCGCAAGATGAAGATGA<br>GTTGCTGCTGTCGTAGTGGTTGTA	239
FD661909	AACAGCTGGAAGAACGCATGGAAG<br>ACAGCTGAAACTTGTTGAACGGGC	200
FD661910	AAGCCAGACATGGGTCCTGAAGAA<br>AGGGCCAGGCGAATAGAGAAACAA	208

FD661912	TGCAGAACACCTGCCTCAAGA	214
	AAGAGAAGAGTACGTAGTGACCGC	
FD661914	AGGAATAACGGGCCATCATGGGTA	245
	TAGTAGAGGTGAATGACGAGAGGCGA	
FD661915	AAATGCCTTCTGGATGGGAACCTG	203
	AAATGAAGCCATCCATGCCACTCC	
FD661916	GGTATTGCTTGCTGCCTGGTTATACG	249
	TAGTCCTCCTCGACGATGGTGTC	
FD661917	ACAACATGTTCCGCTCAATCGTCC	208
	TCTTCCTTCTCCTTGCTGCGTTCA	
FD661919	TCGAGCACTACTGACGACTACGAA	206
	AGATGCTCCTTGTGCTTGTGC	
FD661920	AAGAAGCAGAAGCAGGGTCATCAG	238
	ACAGTAACACGGCTGCAGCAAA	
FD661922	AAGAACGACAAGTGGCACAAGGTG	231
	ATTAACGGATTCAAGACGAGGCGG	
FD661923	TCGGCATTTGTGGTGAACATGGTG	240
	TTGTCAGGCAGCAAGAGTTTCCAC	
FD661924	AAGCTCTGCTGAATGACCCTGTC	233
	AGAGATGGCACTTGGAAGTCTGC	
FD661925	CGATGCTGCATTGGCTGGCTTAATTG	244
	TTGAAAGCAGCAGCACTAGGTCTC	
FD661928	ATGCCATCAAGAAGGATGAAGGAGGG	200
	GAACTGCAGCTGGATCTTCTTCTG	
FD661929	TTCTCTGGTGTGAGAAGTGACAGC	244
	AAGCTTGGACGAGAGCACAGACA	
FD661930	TGGCCAGCCAATGGATTTCAACAC	246
	CTTGTAGTTACACGCCGCAGCAAA	

FD661933	TTGGCGAGCCATTGACACCTCTTA	236
	TTGCTGGATCAAAGCTCCTCCAGA	
FD661936	GCCGTGCAAGTTTATGAAGCCGAA	274
	ATACAGCCAACACACTAGCGACGA	
FD661937	ACAAGGACTCCGGCGACGTCAA	209
	TTCTCCTTCTCCTTCACCTCCGA	
FD661938	ATGTGAAAGCCCTGTACAGAAGGG	217
	ATGTGCTCCACCTTCCTCCATTTC	
FD661940	ATGGTGTGTGGTTTAGGGAGTTGC	257
	ATCACAGACAGCACACCATCTCCA	
FD661941	CCTACTACTCGCTGGGCATCAG	242
	GGGTAGCGCGATATCCAACATGAA	
FD661946	TGCTGAGGTTGGGAGGTTAACGAA	244
	ATCTAACAGCACTGCGAAGGAGCA	
EcActin1 (HE800188)	TTCTCACGCTCAAGTACCCAATCG	237
	AGAGGGAGAGCACTGCTTGAATTG	

Primers used for semi-quantitative RT-PCR analysis of finger miller ESTs

EST name	NCBI GenBank acc#	Primer
Early light inducible protein (ELIP)	FD661789	CAACGGCCGGCTCGCCATGGTGGGC
		TATGCGTGTGTTATACTTGAACTAT
Chlorophyll a/b binding protein	FD661801	GCATCCTCGCGCTCACAAGACAAGT
		ATCTGCGCGCCGGCCTTGAACCACA
RAB17 protein	FD661810	GCAAGACAACCTAGTGATTTGAGTG
		GCCAGTACCCATCTGCTGCTGGTCA
Cytosolic class I small heat shock	FD661843	GGAGAAGGAGGACAAGAACGACAC

protein 3D		CAACAAGCTGATATTTATTCCTTC
Chlorophyll a/b binding protein precursor	FD661878	TTCTCTCTCTCCTGACCACCCCGGG
		GTACCCTGCGGCGCGTTGATGAGCC
Salt stress inducible bZIP protein	FD661884	GGTCCTCTCTGTCGCACAGCCTCGG
		ACCTGGAGCTAATGCTGGAGCAAAA
Fructose bisphosphate aldolase chloroplast precursor, putative, expressed	FD661885	GCTCAACGCCATGAACCAGGGCGCC
		TTCTCTCTCTCCTACAATATTGGCG
Hypothetical protein OsI-003067	FD661886	GGGCGCCGTGCAGATCGAGAAGGTG
		TGCAAGCTTAGAAACTGGTTTTTAG
Late embryogenesis abundant protein D-34 (LEA D-34)	FD661887	CTGAAGCAACACACGACCAGATCAG
		CCACCACCTGTCCCGCGACGAACTC
G-box binding factor 3	FD661896	GGTTACTCAGATCTCAGTAAAAGCA
		ATGCCATTTGGTTGGTTCCATCACT
RNase S-like protein precursor	FD661909	TTTTGATCAGTGTACGTCAATAGAG
		GTAGTCCCTCTCATCGAAGCCGGAG
Fructose-bisphosphate aldolase chloroplast precursor, putative, expressed	FD661912	CTCAACGCCATGAACCAGGGCGCCA
		GTTCTCTCTCTCCTACAATATTGGC

Primers used in qPCR analysis of *AtGBF3*

AtGBF3	CTAGAGTTAAGAACTCAGGAGCTGG
	CCATAACACCAAATTTTCATCTGGAGG
AtEF1α	TCCGTCGGAGCTCAATTCTC
	AGGAAGCTCGAGTGCCAAGTAC

Primers used in PCR confirmation of homozygous *Atgbf3* mutant plants

SALK_082840	AAATGGGAATCCTGAAAATGG

	GAGCATTTCAGTTTGTCCTGC
T-DNA LB	ATTTTGCCGATTTCGGAAC

Primers used to clone orthologs of finger millet drought stress induced genes in maize and other marker gene fragments

ZmHYP	AGACCACGTACCAGCGCGCAC	346
	AGAGCAGCCAAGCAGCGGACAC	
ZmRGAP2	ATGGCCGATGTTGTAGAGCAAG	386
	GGCTCGTGAGAACGGATTTG	
ZmDBH	CAGGCTATGGCATATGGGCC	388
	CACATCTGAGGTTAGCTAGTTGC	
ZmUN	CAAGCCGGTACAGTGCTGG	394
	GTGGAGGTACAATAGCAACAGCG	
ZmGBF3	ATGGGTCATGATGAAGCTGTAG	331
	CCTTGCTGTTCCCAGACTTAGC	
ZmRSLP	GGGTGCTGCAAGCCGACCAC	370
	CCTTGGTGATCGCCTTGAGGC	
ZmUBI7	CAAGCAGCTCGAGGACGGGCGC	490
	ATCCTCCAGCTGCTTGCCAGC	

Other primers used in the study

M13	GTAA AACGACGGCCAGT
	CAGGAAACAGCTATGAC
EcGBF3 GeneRacer primers	CCCTGGTCATGTCCCTCCATTTATG
	CAACTGAATGGCCAGCTAAGGATG
EcGBF3 ectopic expression	ATGGGCCTTGATGAAGCTGTAG
	TCAGCTAGCGGCGATGGCGTCAG
EcGBF3 qPCR in	GGTTACTCAGATCTCAGTAAAAGCA

Arabidopsis transgenic plants	ATGCCATTTGGTTGGTTCCATCACT

Primers used in qPCR analysis of *AtGBF3* predicted targets

AT1G69260	GAGCCACAAGGGAAAGGAAA
	TCTTCTCCACTGCCGTATCT
AT3G29575	CTCACCGGCAGAATTCGTTA
	ACCCAAAGGGTCACAAGAAG
AT1G75170	CTCCAAGCAAATGAACCAAGAC
	GGCAATCTCAGCTCCAGAAA
AT1G72770	ACCTCTCAATGCTTGCTCTAC
	AGCTTCAGGTTCTGGTCTTG
AT5G59220	TGCCAGATAAAGCGTGTGAG
	ACTACGTGTCTCGTCGTAGAT
AtACTIN2	ATTCTTGCTTCCCTCAGCAC
	CCCCAGCTTTTTAAGCCTTT

Table S2. Candidate siRNAs, siRNA efficiency and off targets identified from the siRNA scan tool.

Gene Name	GenBank acc#	DFCI Gene Index	No. of siRNA candidates	siRNA efficiency*	Off targets ID
ZmRGAP2	NM_001139255	TC470875	2	6.1-7.14	TC533739
ZmDBH	NM_001152987	TC459150	4	7.15-8.69	BG462726
ZmUN	NM_001148288	TC488830	5	6.7-8.82	TC539834; TC537690
ZmGBF3	BT063685	TC458346	2	6.44-10.0	Nil
ZmHYP	NM_001158082	TC508811	11	6.49-8.86	CO448659; FL396282; TC494000
ZmRSLP	EU955694	TC490487	18	6.17-9.76	FL097244; TC503176; TC500465; DR821741; TC484715; FL097203; FL455990; FL097212; TC507475; FL011887; FL097197

*Range of 0 to 10 for no silencing to complete silencing of the target.

Table S3. Functional annotation of ESTs identified from finger millet drought stress cDNA library.

NCBI GenBank accession #	Functional annotation	Organism	E-value
FD661788	Putative vacuolar ATP synthase subunit G1	*Zea mays*	1.00E-101
FD661789	Early light-inducible protein	*Panicum virgatum*	7.00E-69
FD661790	Lactoylglutathione lyase family protein	*Zea mays*	4.00E-157
FD661791	Helix-loop-helix-like protein (HLH)	*Zea mays*	8.00E-149
FD661792	17 kDa class I small heat shock protein	*Zea mays*	1.00E-06
FD661793	Putative vacuolar ATP synthase subunit G1	*Zea mays*	5.00E-101
FD661794	Predicted proline rich protein 2	*Zea mays*	5.00E-11
FD661795	Glyoxalase I family protein	*Sorghum bicolor*	7.00E-54
FD661796	Heat shock protein 16.9	*Pennisetum glaucum*	7.00E-29
FD661797	Predicted protein	*Solanum lycopersicum*	6.00E-77
FD661798	Stress kinase	*Brachypodium distachyon*	0
FD661799	Putative heat shock 70 kDa protein	*Oryza sativa Japonica*	2.00E-130
FD661801	Chlorophyll a/b binding protein	*Zea mays*	0
FD661802	Acyl CoA binding protein	*Sorghum bicolor*	3.00E-90
FD661803	Protein of unknown function DUF946	*Gossypium hirsutum*	1.00E-05
FD661804	Subtilisin-like protease SUB5	*Medicago truncatula*	0.099
FD661805	Hypothetical protein	*Solanum lycopersicum*	3.00E-134
FD661806	Osmotic stress-activated protein kinase	*Hordeum vulgare*	0

FD661807	Aminotransferase-like protein	*Oryza sativa Japonica*	0.007
FD661808	Cytochrome P450 superfamily protein	*Brassica rapa*	1.00E-18
FD661809	Hypothetical protein OsJ_000720	*Oryza sativa*	5.00E-35
FD661810	RAB17 protein	*Cleistogenes songorica*	3.00E-160
FD661811	Mitochondrial F1F0 ATP synthase epsilon subunit	*Zea mays*	5.00E-91
FD661812	Collagen, type III, alpha 1	*Medicago truncatula*	0.1
FD661813	Sensor protein baeS	*Medicago truncatula*	9.00E-14
FD661814	Hypothetical protein	*Oryza sativa Japonica*	0.03
FD661815	Hypothetical protein PBAL39_01302	*Raphanus sativus*	8.00E-19
FD661816	Osmotic stress-activated protein kinase	*Oryza sativa Japonica*	0
FD661817	RAB17 protein	*Oryza sativa Japonica*	1.00E-152
FD661818	Photosystem II 10 kDa polypeptide	*Zea mays*	5.00E-86
FD661819	Stress-induced transcription factor NAC1(NAC1)	*Oryza sativa Japonica*	1.00E-87
FD661820	Predicted protein	*Oryza sativa Japonica*	3.00E-30
FD661821	Dehydrin1	*Cleistogenes songorica*	6.00E-87
FD661822	GNAS complex locus	*Zea mays*	3.00E-20
FD661823	Nuclear ribonucleoprotein-associated protein B	*Vitis vinifera*	0.008
FD661824	Proline rich protein	*Solanum lycopersicum*	2.00E-112
FD661825	Osmotic stress-activated protein kinase	*Zea mays*	0
FD661826	RAB21 protein	*Oryza sativa Japonica*	7.00E-75

FD661827	Putative 4-hydroxyphenylpyruvate dioxygenase	*Zea mays*	0
FD661829	Putative arginine/serine-rich protein	*Panicum virgatum*	3.00E-05
FD661830	Chain D, 4-Hydroxyphenylpyruvate Dioxygenase	*Oryza sativa Japonica*	1.00E-67
FD661831	Osmotic stress-activated protein kinase	*Hordeum vulgare*	0
FD661832	Cytokinesis protein sepA	*Vitis vinifera*	0.091
FD661833	Synptobrevin-related protein	*Oryza sativa Japonica*	0
FD661834	DNAJ heat shock N-terminal domain-containing protein	*Brachypodium distachyon*	8.00E-154
FD661836	Alpha 1 type XXIV collagen	*Solanum lycopersicum*	0.095
FD661837	Hypothetical protein CIMG_06839	*Zea mays*	1.00E-13
FD661838	Hypothetical protein OsJ_032036	*Solanum lycopersicum*	0.028
FD661839	Single stranded DNA binding protein	*Zea mays*	7.00E-04
FD661840	Hypothetical protein	*Hordeum vulgare*	2.00E-38
FD661841	Osmotic stress-activated protein kinase	*Brachypodium distachyon*	0
FD661842	Heat shock protein 17.0	*Oryza sativa Japonica*	8.00E-104
FD661843	Cytosolic class I small heat shock protein 3D	*Oryza sativa Japonica*	2.00E-59
FD661844	Rho GTPase activating protein 2	*Sorghum bicolor*	0
FD661845	60S ribosomal protein L10	*Zea mays*	0
FD661846	Splicing factor, arginine/serine-rich 4	*Hordeum vulgare*	5.00E-141
FD661847	Alpha 1 type IX collagen chain	*Zea mays*	3.00E-08
FD661848	18 kDa heat shock protein	*Zea mays*	2.00E-20

FD661849	Hypothetical protein RUMTOR_0292	*Populus trichocarpa*	0.1
FD661850	Hypothetical protein PB404290.00.0	*Oryza sativa Japonica*	9.00E-12
FD661851	Translation initiation factor IF-2	*Zea mays*	4.00E-95
FD661852	Enolase1	*Oryza sativa Japonica*	8.00E-109
FD661853	Dehydrin	*Sorghum bicolor*	3.00E-53
FD661854	FtsH protease 1	*Oryza sativa Japonica*	7.00E-174
FD661855	Proline-rich protein-1	*Brachypodium distachyon*	1.00E-13
FD661856	Rab	*Cleistogenes songorica*	6.00E-61
FD661857	Predicted protein	*Arachis hypogaea*	3.00E-11
FD661858	Retrotransposon protein, putative, Ty1-copia subclass	*Solanum lycopersicum*	0
FD661859	Hypothetical protein OsI_015128	*Zea mays*	5.00E-25
FD661860	Cold stable pyruvate, orthophosphate dikinase	*Sorghum bicolor*	5.00E-121
FD661861	Voltage-gated potassium channel subunit Kv10.1b	*Brachypodium distachyon*	0.007
FD661862	Multiple stress-responsive zinc-finger protein (ZF_1)	*Hordeum vulgare*	5.00E-07
FD661863	Photosystem II 10 kDa polypeptide	*Zea mays*	1.00E-122
FD661864	Similar to mCG145496	*Oryza sativa Japonica*	0.086
FD661865	MT-like protein	*Cynodon dactylon*	8.00E-158
FD661866	Similar to RISBZ4	*Zea mays*	5.00E-141
FD661867	Actin-related protein 3	*Zea mays*	0
FD661868	RAB17 protein	*Oryza sativa Japonica*	2.00E-85

FD661869	Putative ethylene-forming enzyme	*Sorghum bicolor*	7.00E-95
FD661870	Hypothetical protein OsI_024391	*Oryza sativa Japonica*	9.00E-94
FD661871	Pre-rRNA-processing protein ESF2	*Vitis vinifera*	3.00E-20
FD661872	Metallothionein-like protein	*Brachypodium distachyon*	8.00E-40
FD661873	Early drought induced protein	*Hordeum vulgare*	6.00E-46
FD661875	DEAD/DEAH box helicase	*Sorghum bicolor*	0
FD661876	Salt tolerance protein 2	*Sorghum bicolor*	4.00E-102
FD661877	Putative proteinase inhibitor	*Zea mays*	1.00E-06
FD661878	Chlorophyll a/b-binding protein precursor	*Zea mays*	0
FD661879	Glutaredoxin	*Zea mays*	1.00E-111
FD661880	Unknown protein	*Zea mays*	8.00E-149
FD661881	WW domain binding protein 11	*Zea mays*	4.00E-13
FD661883	Putative Acyl-CoA binding protein	*Oryza sativa Japonica*	7.00E-85
FD661884	Salt-stress inducible bZIP protein (bZIP)	*Zea mays*	8.00E-109
FD661887	Late embryogenesis abundant protein D-34	*Hordeum vulgare*	2.00E-86
FD661890	Rice homologue of Tat binding protein	*Oryza sativa Japonica*	1.00E-172
FD661893	Anthocyanidin reductase	*Zea mays*	1.00E-75
FD661894	Phosphoenolpyruvate carboxylase	*Oryza sativa*	3.00E-78
FD661895	Photosystem I complex PsaH subunit precursor	*Sorghum bicolor*	1.00E-136
FD661896	G-box binding factor 3 (GBF3)	*Oryza sativa Japonica*	2.00E-99

FD661897	Non-specific lipid-transfer protein	*Zea mays*	6.00E-49
FD661900	Nuclear antigen	*Sorghum bicolor*	1.00E-96
FD661901	Metallothionein-like protein 3B	*Brachypodium distachyon*	1.00E-41
FD661902	RAB17 protein	*Oryza sativa Japonica*	2.00E-81
FD661903	Zinc finger (B-box type) family protein (ZF_2)	*Zea mays*	2.00E-114
FD661904	Translation initiation factor eIF1	*Zea mays*	8.00E-169
FD661907	Putative cyanase	*Brachypodium distachyon*	1.00E-66
FD661908	Geranylgeranyl hydrogenase	*Zea mays*	0
FD661909	RNase S-like protein precursor	*Hordeum vulgare*	3.00E-34
FD661910	Proteasome subunit beta type 3	*Brachypodium distachyon*	0
FD661911	RAB17 protein	*Sorghum bicolor*	3.00E-58
FD661912	Fructose-bisphosphate aldolase, chloroplast precursor	*Zea mays*	2.00E-68
FD661913	Similar to mCG145496	*Populus trichocarpa*	0.09
FD661914	Extracellular matrix protein	*Glycine max*	3.00E-65
FD661915	Oxidoreductase	*Zea mays*	3.00E-118
FD661916	Hypothetical protein OsI_023315	*Brachypodium distachyon*	3.00E-13
FD661917	Heat shock protein 17.2	*Oryza sativa Japonica*	1.00E-131
FD661918	ATP-binding region, ATPase domain protein	*Sorghum bicolor*	2.00E-13
FD661919	Drought inducible 22 kDa protein	*Saccharum officinarum*	1.00E-62
FD661920	Predicted: similar to type X collagen	*Zea mays*	2.00E-26

FD661921	Putative proteinase inhibitor	*Zea mays*	1.00E-06
FD661922	Chaperone	*Oryza sativa Japonica*	3.00E-63
FD661923	Chain A, Pyruvate Phosphate Dikinase	*Brachypodium distachyon*	1.00E-82
FD661924	Asorbate peroxidase	*Pennisetum glaucum*	8.00E-80
FD661925	Heparan sulfate 6-O-sulfotransferase CG12249-PA, isoform A	*Zea mays*	2.00E-66
FD661926	Hypothetical protein MED193_13008	*Vitis vinifera*	2.2
FD661928	Hypothetical protein OsI_024391	*Oryza sativa Japonica*	2.00E-65
FD661929	Saposin-like type B, region 1 family protein	*Oryza sativa Japonica*	2.00E-53
FD661930	Predicted proline-rich protein	*Hordeum vulgare*	3.00E-38
FD661931	Actin-related protein 3	*Brachypodium distachyon*	0
FD661933	Putative plastidic ATP/ADP transporter	*Glycine max*	4.00E-72
FD661935	DEAD/DEAH box helicase	*Brachypodium distachyon*	6.00E-155
FD661936	Thioredoxin H	*Zea mays*	7.00E-16
FD661937	Late embryogenesis abundant protein	*Sorghum bicolor*	5.00E-96
FD661938	Peptidylprolyl isomerase	*Oryza sativa Japonica*	1.00E-31
FD661940	ATP-binding region, ATPase domain protein	*Glycine max*	0.01
FD661941	Unknown protein	*Sorghum bicolor*	3.00E-163
FD661942	Predicted protein	*Zea mays*	1.6
FD661945	Hypothetical protein LbrM03_V2.0300	*Oryza sativa Japonica*	0.004
FD661947	Type 1 metallothionein-like protein	*Cynodon dactylon*	2.00E-115

FD661948	Predicted protein	*Carica papaya*	3.9
FD661788	Putative vacuolar ATP synthase subunit G1	*Zea mays*	1.00E-101
FD661789	Early light-inducible protein	*Panicum virgatum*	7.00E-69
FD661790	Lactoylglutathione lyase family protein	*Zea mays*	4.00E-157
FD661791	Helix-loop-helix-like protein (HLH)	*Zea mays*	8.00E-149
FD661792	17 kDa class I small heat shock protein	*Zea mays*	1.00E-06
FD661793	Putative vacuolar ATP synthase subunit G1	*Zea mays*	5.00E-101
FD661794	Predicted proline rich protein 2	*Zea mays*	5.00E-11
FD661795	Glyoxalase I family protein	*Sorghum bicolor*	7.00E-54
FD661796	Heat shock protein 16.9	*Pennisetum glaucum*	7.00E-29
FD661797	Predicted protein	*Solanum lycopersicum*	6.00E-77
FD661798	Stress kinase	*Brachypodium distachyon*	0
FD661799	Putative heat shock 70 kDa protein	*Oryza sativa Japonica*	2.00E-130
FD661801	Chlorophyll a/b binding protein	*Zea mays*	0
FD661802	Acyl CoA binding protein	*Sorghum bicolor*	3.00E-90
FD661803	Protein of unknown function DUF946	*Gossypium hirsutum*	1.00E-05
FD661804	Subtilisin-like protease SUB5	*Medicago truncatula*	0.099
FD661805	Hypothetical protein	*Solanum lycopersicum*	3.00E-134

Table S4. Validation of drought stress response of EST's by expression analysis.

NCBI GenBank accession #	Functional annotation	60% FC	35% FC	60% FC recovery	35% FC recovery
FD661788	Putative vacuolar ATP synthase subunit G1	0.55	3.24	0.97	0.24
FD661789	Early light-inducible protein	160.17	121.09	23.65	6.98
FD661790	Lactoylglutathione lyase family protein	0.70	10.78	1.70	0.99
FD661791	Helix-loop-helix-like protein (HLH)	0.98	2.39	1.73	1.10
FD661792	17 kDa class I small heat shock protein	0.12	0.47	0.26	0.01
FD661795	Glyoxalase I family protein	0.60	12.02	0.22	0.10
FD661798	Stress kinase	0.71	4.60	1.84	1.37
FD661799	Putative heat shock 70 kDa protein	0.38	2.65	1.18	0.27
FD661801	Chlorophyll a/b binding protein	0.09	0.04	0.26	0.05
FD661802	Acyl CoA binding protein	1.12	1.31	0.42	2.32
FD661805	Hypothetical protein	0.04	0.05	0.30	0.10
FD661806	Osmotic stress-activated protein kinase	0.28	3.75	1.31	2.06
FD661807	Aminotransferase-like protein	0.16	0.12	0.59	1.04
FD661809	Hypothetical protein OsJ_000720	0.83	13.83	0.40	0.10
FD661810	RAB17 protein	113.96	125.99	107.53	117.40
FD661811	Mitochondrial F1F0 ATP synthase epsilon subunit	0.35	1.86	0.56	0.48
FD661816	Osmotic stress-activated protein kinase	0.72	4.57	2.02	1.70
FD661818	Photosystem II 10 kDa polypeptide	0.17	0.13	0.78	0.03
FD661819	Stress-induced transcription factor NAC1(NAC1)	0.51	3.39	0.06	0.09
FD661820	Predicted protein	0.11	1.10	0.17	0.10
FD661824	Proline rich protein	0.32	3.40	0.41	0.15
FD661827	Putative 4-hydroxyphenylpyruvate dioxygenase	0.21	2.87	0.11	0.23
FD661830	Chain D, 4-Hydroxyphenylpyruvate Dioxygenase	0.15	17.85	0.29	0.09
FD661833	Synptobrevin-related protein	0.56	2.58	2.53	1.91
FD661834	DNAJ heat shock N-terminal domain-containing protein	0.05	0.50	0.32	0.06
FD661839	Single stranded DNA binding protein	0.08	0.46	0.48	0.04
FD661843	Cytosolic class I small heat shock protein 3D	2.18	113.02	0.47	1.84
FD661844	Rho GTPase activating protein 2	13.62	1.36	7.45	12.03

FD661845	60S ribosomal protein L10	0.41	1.26	0.94	2.84
FD661851	Translation initiation factor IF-2	0.26	1.53	1.41	1.21
FD661852	Enolase1	0.03	0.05	0.33	0.13
FD661854	FtsH protease 1	0.64	1.61	0.26	0.61
FD661860	Cold stable pyruvate, orthophosphate dikinase	0.22	0.12	0.28	0.66
FD661861	Voltage-gated potassium channel subunit Kv10.1b	0.35	0.08	0.02	0.37
FD661862	Multiple stress-responsive zinc-finger protein (ZF_1)	0.96	1.76	0.50	0.66
FD661865	MT-like protein	0.21	1.06	0.73	0.29
FD661866	Similar to RISBZ4	0.15	0.82	0.55	0.26
FD661867	Actin-related protein 3	6.34	13.48	2.39	8.01
FD661869	Putative ethylene-forming enzyme	0.72	5.05	0.71	1.28
FD661870	Hypothetical protein OsI_024391	2.56	21.61	0.92	1.12
FD661871	Pre-rRNA-processing protein ESF2	0.05	4.27	0.48	0.17
FD661872	Metallothionein-like protein	1.97	6.19	1.81	5.11
FD661873	Early drought induced protein	2.80	17.70	0.57	2.61
FD661875	DEAD/DEAH box helicase	5.16	1.76	3.53	15.90
FD661876	Salt tolerance protein 2	1.07	2.15	1.41	4.55
FD661877	Putative proteinase inhibitor	1.58	34.51	1.07	3.04
FD661878	Chlorophyll a/b-binding protein precursor	0.10	0.03	0.24	0.03
FD661879	Glutaredoxin	1.43	2.47	0.58	1.78
FD661880	Unknown protein	24.31	45.05	1.32	9.64
FD661883	Putative Acyl-CoA binding protein	1.03	1.41	1.15	4.37
FD661884	Salt-stress inducible bZIP protein (bZIP)	27.92	92.05	0.54	0.48
FD661885	Fructose-bisphosphate aldolase, chloroplast precursor	0.05	0.05	0.13	0.13
FD661886	Hypothetical protein OsI_003067	2.42	95.41	0.07	0.09
FD661887	Late embryogenesis abundant protein D-34	0.25	9.04	0.14	0.06
FD661890	Rice homologue of Tat binding protein	3.20	5.06	1.28	8.25
FD661892	Salt tolerance protein 1	0.51	2.05	1.62	1.62
FD661893	Anthocyanidin reductase	0.67	1.73	0.50	1.25
FD661894	Phosphoenolpyruvate carboxylase	0.24	0.13	0.34	0.29
FD661895	Photosystem I complex PsaH subunit precursor	0.60	0.02	0.19	0.13

FD661896	G-box binding factor 3 (GBF3)	45.90	76.41	0.64	1.91
FD661897	Non-specific lipid-transfer protein	0.84	2.06	0.58	0.14
FD661900	Nuclear antigen	0.28	1.49	3.82	0.79
FD661903	Zinc finger (B-box type) family protein (ZF_2)	0.01	0.15	0.22	0.02
FD661904	Translation initiation factor eIF1	0.79	5.85	1.28	0.21
FD661907	Putative cyanase	0.18	0.58	0.62	0.04
FD661908	Geranylgeranyl hydrogenase	0.08	0.04	4.17	0.12
FD661909	RNase S-like protein precursor	16.11	1.66	74.14	0.13
FD661910	Proteasome subunit beta type 3	0.43	2.17	0.76	0.45
FD661912	Fructose-bisphosphate aldolase, chloroplast precursor	0.02	0.04	0.26	0.01
FD661914	Extracellular matrix protein	0.67	4.46	1.20	1.33
FD661915	Oxidoreductase	0.87	2.63	1.51	0.47
FD661916	Hypothetical protein OsI_023315	0.24	0.51	2.55	0.01
FD661917	Heat shock protein 17.2	0.38	4.11	2.15	3.67
FD661919	Drought inducible 22 kDa protein	12.80	21.19	0.39	0.21
FD661920	Predicted: similar to type X collagen	0.39	2.01	2.25	0.21
FD661922	Chaperone	0.57	4.43	0.48	0.28
FD661923	Chain A, Pyruvate Phosphate Dikinase	0.07	0.09	0.34	0.11
FD661924	Asorbate peroxidase	0.99	4.97	1.85	0.26
FD661925	Heparan sulfate 6-O-sulfotransferase CG12249-PA, isoform A	0.17	0.95	0.39	0.35
FD661928	Hypothetical protein OsI_024391	0.93	18.83	1.46	0.24
FD661929	Saposin-like type B, region 1 family protein	0.71	5.99	3.75	0.53
FD661930	Predicted proline-rich protein	0.39	2.98	0.62	0.24
FD661933	Putative plastidic ATP/ADP transporter	3.90	31.02	14.23	10.57
FD661936	Thioredoxin H	0.23	0.84	1.03	0.99
FD661937	Late embryogenesis abundant protein	0.09	1.70	0.10	0.03
FD661938	Peptidylprolyl isomerase	0.16	0.69	0.79	0.30
FD661940	ATP-binding region, ATPase domain protein	0.21	0.62	1.87	0.40
FD661941	Unknown protein	0.13	0.23	0.30	0.17
FD661946	ATP-binding region, ATPase domain protein	0.15	1.07	0.72	0.11

*The expression values were normalized to *EcActin* expression levels and the relative change in transcript levels compared to well-watered condition was calculated. The expression values between 0-1, 1.01-2, 2.01-5 and >5.01 are colored in yellow, blue, green and red, respectively.

Table S5: The expression targets of GBF3 predicted from a regulatory network (q vlaue ≥0.001)

**Target**	**Description**	**Z Score**
AT1G69260	ABI FIVE BINDING PROTEIN (AFP1)	6.071
AT3G29575	ABI FIVE BINDING PROTEIN 3 (AFP3)	5.559
AT1G75170	Sec14p-like phosphatidylinositol transfer family protein	5.539
AT1G72770	HYPERSENSITIVE TO ABA1 (HAB1)	5.225
AT5G59220	HIGHLY ABA-INDUCED PP2C GENE 1 (HAI1)	5.203
AT2G40830	RING-H2 FINGER C1A (RHC1A)	4.772
AT3G11420	beta-1,3-N-acetylglucosaminyltransferase lunatic protein, putative (DUF604)	4.753
AT3G62260	Protein phosphatase 2C family protein	4.661
AT1G77450	NAC DOMAIN CONTAINING PROTEIN 32 (NAC032)	4.626
AT3G51890	CLATHRIN LIGHT CHAIN 3 (CLC3)	4.608
AT5G19875	transmembrane protein	4.517
AT3G09910	RAB GTPASE HOMOLOG C2B (RABC2b)	4.516
AT5G57050	ABA INSENSITIVE 2 (ABI2)	4.474
AT1G64660	METHIONINE GAMMA-LYASE (MGL)	4.473
AT4G31860	Protein phosphatase 2C family protein	4.464
AT2G26600	Glycosyl hydrolase superfamily protein	4.45
AT4G22820	STRESS ASSOCIATED PROTEIN 9 (SAP9)	4.424
AT5G24530	DOWNY MILDEW RESISTANT 6 (DMR6)	4.415
AT1G67360	LD-ASSOCIATED PROTEIN 1 (LDAP1)	4.411
AT1G04120	ATP-BINDING CASSETTE C5 (ABCC5)	4.41
AT1G01720	NAC DOMAIN CONTAINING PROTEIN 32 (NAC032)	4.393
AT3G20250	PUMILIO 5 (PUM5)	4.358
AT1G07870	Protein kinase superfamily protein	4.322
AT3G12580	HEAT SHOCK PROTEIN 70 (HSP70)	4.318
AT5G42050	DCD (Development and Cell Death) domain protein	4.309
AT5G11650	alpha/beta-Hydrolases superfamily protein; MAGL13	4.295
AT1G22930	T-complex protein 11	4.28
AT3G17800	MEB5.2	4.268

AT2G33590	CCR(CINNAMOYL COA:NADP OXIDOREDUCTASE)-LIKE 1 (CRL1)	4.266
AT4G29950	Ypt/Rab-GAP domain of gyp1p superfamily protein	4.257
AT2G37970	SOUL-1	4.254
AT1G21080	DNAJ heat shock N-terminal domain-containing protein	4.246
AT1G05170	Galactosyltransferase family protein	4.229
AT5G22000	RING-H2 GROUP F2A (RHF2A)	4.228
AT1G06570	PHYTOENE DESATURATION 1 (PDS1)	4.182
AT3G52800	A20/AN1-like zinc finger family protein	4.175
AT1G60190	PLANT U-BOX 19 (PUB19)	4.166
AT3G17000	UBIQUITIN-CONJUGATING ENZYME 32 (UBC32)	4.164
AT5G06960	OCS-ELEMENT BINDING FACTOR 5 (OBF5)	4.157
AT4G34000	ABSCISIC ACID RESPONSIVE ELEMENTS-BINDING FACTOR 3 (ABF3)	4.128
AT5G07070	CBL-INTERACTING PROTEIN KINASE 2 (CIPK2)	4.112
AT4G05050	UBIQUITIN 11 (UBQ11)	4.098
AT3G51000	alpha/beta-Hydrolases superfamily protein	4.097
AT3G07700	SALT-INDUCED ABC1 KINASE 1 (SIA1)	4.096
AT5G62570	CALMODULIN-BINDING PROTEIN 60A (CBP60A)	4.09
AT3G04240	SECRET AGENT (SEC)	4.055
AT4G01280	Homeodomain-like superfamily protein	4.03
AT3G23920	BETA-AMYLASE 1 (BAM1)	4.019
AT2G20560	DNAJ heat shock family protein	4.009
AT1G68620	alpha/beta-Hydrolases superfamily protein	4.008
AT4G17840	FUNCTIONS IN: molecular_function unknown	4.005
AT1G79270	EVOLUTIONARILY CONSERVED C-TERMINAL REGION 8 (ECT8)	4.005
AT5G64930	CONSTITUTIVE EXPRESSION OF PR GENES 5 (CPR5)	3.997
AT5G04410	NAC DOMAIN CONTAINING PROTEIN 2 (NAC2)	3.983
AT5G55860	Plant protein of unknown function (DUF827)	3.976
AT4G30490	AFG1-like ATPase family protein	3.965
AT1G17440	ENHANCED ETHYLENE RESPONSE 4 (EER4)	3.958
AT2G16720	MYB DOMAIN PROTEIN 7 (MYB7)	3.954
AT3G54680	proteophosphoglycan-related	3.949

AT2G43320	S-adenosyl-L-methionine-dependent methyltransferases superfamily protein	3.927
AT2G17220	KINASE 3 (KIN3)	3.912
AT1G75400	RING/U-box superfamily protein	3.911
AT5G10740	Protein phosphatase 2C family protein	3.9
AT3G57880	Calcium-dependent lipid-binding (CaLB domain) plant phosphoribosyltransferase family protein	3.887
AT3G24500	MULTIPROTEIN BRIDGING FACTOR 1C (MBF1C)	3.886
AT4G30470	NAD(P)-binding Rossmann-fold superfamily protein	3.885
AT3G24180	Beta-glucosidase, GBA2 type family protein	3.88
AT2G46080	BYPASS 2 (BPS2)	3.879
AT4G34230	CINNAMYL ALCOHOL DEHYDROGENASE 5 (CAD5)	3.867
AT3G01590	Galactose mutarotase-like superfamily protein	3.865
AT3G61890	HOMEOBOX 12 (HB-12)	3.855
AT3G06760	Drought-responsive family protein	3.844
AT5G58720	smr (Small MutS Related) domain-containing protein	3.843
AT3G16940	calmodulin binding	3.83
AT5G54840	(SGP1)	3.83
AT1G74310	HEAT SHOCK PROTEIN 101 (HSP101)	3.825
AT1G70590	F-box family protein	3.82
AT4G02890	(UBQ14)	3.816
AT1G03080	NETWORKED 1D (NET1D)	3.815
AT1G63840	RING/U-box superfamily protein	3.813
AT3G53780	RHOMBOID-LIKE PROTEIN 4 (RBL4)	3.813
AT2G35940	BEL1-LIKE HOMEODOMAIN 1 (BLH1)	3.811
AT4G17530	RAB GTPASE HOMOLOG 1C (RAB1C)	3.809
AT1G03290	unknown protein	3.805
AT5G22630	AROGENATE DEHYDRATASE 5 (ADT5)	3.805
AT4G37790	(HAT22)	3.804
AT5G01850	Protein kinase superfamily protein	3.799
AT5G57910	unknown protein	3.799
AT5G53130	CYCLIC NUCLEOTIDE GATED CHANNEL 1 (CNGC1)	3.799
AT1G67920	unknown protein	3.785

AT1G30640	Protein kinase family protein	3.779
AT3G53810	L-TYPE LECTIN RECEPTOR KINASE IV.2 (LECRK-IV.2)	3.774
AT5G37540	Eukaryotic aspartyl protease family protein	3.77
AT1G75810	unknown protein	3.759
AT4G38060	CLAVATA COMPLEX INTERACTOR 2 (CCI2)	3.759
AT3G19290	ABRE BINDING FACTOR 4 (ABF4)	3.755
AT3G44100	MD-2-related lipid recognition domain-containing protein	3.754
AT2G31350	GLYOXALASE 2-5 (GLX2-5)	3.754
AT2G46680	HOMEOBOX 7 (HB-7)	3.747
AT4G11350	Protein of unknown function (DUF604)	3.736
AT5G24870	RING/U-box superfamily protein	3.736
AT1G32230	RADICAL-INDUCED CELL DEATH1 (RCD1)	3.735
AT5G64510	TUNICAMYCIN INDUCED 1 (TIN1)	3.725
AT2G30550	DAD1-LIKE LIPASE 3 (DALL3)	3.723
AT3G03310	LECITHIN:CHOLESTEROL ACYLTRANSFERASE 3 (LCAT3)	3.714
AT5G11970	Protein of unknown function (DUF3511)	3.704
AT1G55530	RING/U-box superfamily protein	3.699
AT2G28400	Protein of unknown function, DUF584	3.692
AT3G63010	GA INSENSITIVE DWARF1B (GID1B)	3.686
AT2G31130	unknown protein	3.684
AT1G26690	emp24/gp25L/p24 family/GOLD family protein	3.682
AT1G01550	BYPASS 1 (BPS1)	3.678
AT2G26170	CYTOCHROME P450, FAMILY 711, SUBFAMILY A, POLYPEPTIDE 1 (CYP711A1)	3.678
AT3G11410	PROTEIN PHOSPHATASE 2CA (PP2CA)	3.674
AT5G07920	DIACYLGLYCEROL KINASE1 (DGK1)	3.673
AT1G29330	ENDOPLASMIC RETICULUM RETENTION DEFECTIVE 2 (ERD2)	3.672
AT3G08720	SERINE/THREONINE PROTEIN KINASE 2 (S6K2)	3.672
AT3G46450	SEC14 cytosolic factor family protein / phosphoglyceride transfer family protein	3.67
AT5G52640	HEAT SHOCK PROTEIN 90.1 (HSP90.1)	3.668
AT5G11090	serine-rich protein-related	3.667
AT2G30360	SOS3-INTERACTING PROTEIN 4 (SIP4)	3.665

AT1G21450	SCARECROW-LIKE 1 (SCL1)	3.664
AT3G10250	Plant protein 1589 of unknown function	3.66
AT5G46410	SCP1-LIKE SMALL PHOSPHATASE 4 (SSP4)	3.652
AT5G35320	unknown protein	3.65
AT5G58380	SOS3-INTERACTING PROTEIN 1 (SIP1)	3.65
AT2G24100	ALTERED SEED GERMINATION 1 (ASG1)	3.646
AT2G40270	Protein kinase family protein	3.645
AT1G49710	FUCOSYLTRANSFERASE 12 (FUT12)	3.645
AT3G05830	(ATNEAP1)	3.641
AT5G42990	UBIQUITIN-CONJUGATING ENZYME 18 (UBC18)	3.639
AT2G46800	ZINC TRANSPORTER OF ARABIDOPSIS THALIANA (ZAT)	3.637
AT3G19580	ZINC-FINGER PROTEIN 2 (ZF2)	3.635
AT1G67880	beta-1,4-N-acetylglucosaminyltransferase family protein	3.632
AT3G57530	CALCIUM-DEPENDENT PROTEIN KINASE 32 (CPK32)	3.631
AT2G47180	GALACTINOL SYNTHASE 1 (GolS1)	3.631
AT5G23850	FUNCTIONS IN: molecular_function unknown	3.624
AT5G58350	WITH NO LYSINE (K) KINASE 4 (WNK4)	3.624
AT5G57900	SKP1 INTERACTING PARTNER 1 (SKIP1)	3.61
AT2G44970	alpha/beta-Hydrolases superfamily protein	3.606
AT1G09140	SERINE/ARGININE-RICH PROTEIN SPLICING FACTOR 30 (SR30)	3.605
AT1G76970	Target of Myb protein 1	3.6
AT5G53120	SPERMIDINE SYNTHASE 3 (SPDS3)	3.594
AT5G45130	RAB HOMOLOG 1 (RHA1)	3.592
AT3G10800	(BZIP28)	3.591
AT4G35110	Arabidopsis phospholipase-like protein (PEARLI 4) family	3.59
AT1G07350	SERINE/ARGININE RICH-LIKE PROTEIN 45A (SR45a)	3.588
AT3G15430	Regulator of chromosome condensation (RCC1) family protein	3.584
AT2G29990	ALTERNATIVE NAD(P)H DEHYDROGENASE 2 (NDA2)	3.584
AT4G05020	NAD(P)H DEHYDROGENASE B2 (NDB2)	3.58
AT2G30490	CINNAMATE-4-HYDROXYLASE (C4H)	3.578
AT2G34730	myosin heavy chain-related	3.574

AT5G22290	NAC DOMAIN CONTAINING PROTEIN 89 (NAC089)	3.574
AT5G61820	FUNCTIONS IN: molecular_function unknown	3.573
AT1G80820	CINNAMOYL COA REDUCTASE (CCR2)	3.572
AT5G16830	SYNTAXIN OF PLANTS 21 (SYP21)	3.57
AT2G21940	SHIKIMATE KINASE 1 (SK1)	3.56
AT1G31130	unknown protein	3.559
AT1G48320	DHNA-COA THIOESTERASE 1 (DHNAT1)	3.559
AT4G27780	ACYL-COA BINDING PROTEIN 2 (ACBP2)	3.554
AT5G47120	BAX INHIBITOR 1 (BI1)	3.553
AT3G03790	ankyrin repeat family protein / regulator of chromosome condensation (RCC1) family protein	3.551
AT5G52530	dentin sialophosphoprotein-related	3.55
AT1G01650	SIGNAL PEPTIDE PEPTIDASE-LIKE 4 (SPPL4)	3.545
AT3G57540	REMORIN GROUP 4 1 (REM4.1)	3.544
AT1G76040	CALCIUM-DEPENDENT PROTEIN KINASE 29 (CPK29)	3.543
AT4G37460	SUPPRESSOR OF RPS4-RLD 1 (SRFR1)	3.542
AT1G52080	(AR791)	3.538
AT1G32870	NAC DOMAIN PROTEIN 13 (NAC13)	3.529
AT3G56310	Melibiase family protein	3.527
AT3G16990	Haem oxygenase-like, multi-helical	3.526
AT1G61660	(ATBHLH112)	3.524
AT4G12400	HOP3 (Hop3)	3.521
AT2G33380	RESPONSIVE TO DESICCATION 20 (RD20)	3.52
AT1G74360	Leucine-rich repeat protein kinase family protein	3.518
AT2G46500	PHOSPHOINOSITIDE 4-KINASE GAMMA 4 (PI4K GAMMA 4)	3.514
AT4G23630	VIRB2-INTERACTING PROTEIN 1 (BTI1)	3.511
AT1G17550	HOMOLOGY TO ABI2 (HAB2)	3.508
AT5G16880	Target of Myb protein 1	3.503
AT3G05210	(ERCC1)	3.503
AT4G14220	RING-H2 GROUP F1A (RHF1A)	3.503
AT1G09070	SOYBEAN GENE REGULATED BY COLD-2 (SRC2)	3.501
AT5G62020	HEAT SHOCK TRANSCRIPTION FACTOR B2A (HSFB2A)	3.5

AT5G54870	unknown protein	3.499
AT5G03490	UDP GLYCOSYLTRANSFERASE 89A2 (UGT89A2)	3.497
AT1G23440	Peptidase C15, pyroglutamyl peptidase I-like	3.496
AT5G24810	ABC1 family protein	3.493
AT4G28390	ADP/ATP CARRIER 3 (AAC3)	3.492
AT5G13820	TELOMERIC DNA BINDING PROTEIN 1 (TBP1)	3.489
AT4G30210	P450 REDUCTASE 2 (ATR2)	3.487
AT1G80610	unknown protein	3.481
AT4G34150	Calcium-dependent lipid-binding (CaLB domain) family protein	3.481
AT1G64060	RESPIRATORY BURST OXIDASE PROTEIN F (RBOH F)	3.476
AT5G09620	Octicosapeptide/Phox/Bem1p family protein	3.47
AT1G56660	unknown protein	3.469
AT3G12740	ALA-INTERACTING SUBUNIT 1 (ALIS1)	3.469
AT3G55640	Mitochondrial substrate carrier family protein	3.464
AT5G11110	SUCROSE PHOSPHATE SYNTHASE 2F (SPS2F)	3.461
AT5G13200	GEM-RELATED 5 (GER5)	3.458
AT4G21580	oxidoreductase, zinc-binding dehydrogenase family protein	3.457
AT4G24100	Protein kinase superfamily protein	3.455
AT1G42990	BASIC REGION/LEUCINE ZIPPER MOTIF 60 (BZIP60)	3.454
AT5G12030	HEAT SHOCK PROTEIN 17.6A (HSP17.6A)	3.448
AT2G37760	ALDO-KETO REDUCTASE FAMILY 4 MEMBER C8 (AKR4C8)	3.444
AT4G16760	ACYL-COA OXIDASE 1 (ACX1)	3.44
AT5G45410	unknown protein	3.435
AT1G23040	hydroxyproline-rich glycoprotein family protein	3.434
AT1G69610	FUNCTIONS IN: structural constituent of ribosome	3.434
AT2G41190	Transmembrane amino acid transporter family protein	3.434
AT4G23850	LONG-CHAIN ACYL-COA SYNTHETASE 4 (LACS4)	3.434
AT5G56980	unknown protein	3.43
AT2G33580	LYSM-CONTAINING RECEPTOR-LIKE KINASE 5 (LYK5)	3.426
AT4G29330	DERLIN-1 (DER1)	3.424
AT4G00500	alpha/beta-Hydrolases superfamily protein	3.423

AT2G27580	STRESS ASSOCIATED PROTEIN3 (SAP3)	3.423
AT4G29160	(SNF7.1)	3.416
AT1G55110	INDETERMINATE(ID)-DOMAIN 7 (IDD7)	3.413
AT5G21090	Leucine-rich repeat (LRR) family protein	3.409
AT1G68910	WPP DOMAIN-INTERACTING PROTEIN 2 (WIT2)	3.408
AT5G15860	PRENYLCYSTEINE METHYLESTERASE (PCME)	3.407
AT5G63370	CYCLIN-DEPENDENT KINASE G1 (CDKG1)	3.395
AT4G34390	EXTRA-LARGE GTP-BINDING PROTEIN 2 (XLG2)	3.393
AT5G54110	MEMBRANE-ASSOCIATED MANNITOL-INDUCED (MAMI)	3.392
AT5G51070	EARLY RESPONSIVE TO DEHYDRATION 1 (ERD1)	3.391
AT1G21000	PLATZ transcription factor family protein	3.389
AT4G23570	(SGT1A)	3.388
AT4G26080	ABA INSENSITIVE 1 (ABI1)	3.388
AT4G23050	PAS domain-containing protein tyrosine kinase family protein	3.386
AT1G25370	Protein of unknown function (DUF1639)	3.381
AT2G18090	PHD finger family protein / SWIB complex BAF60b domain-containing protein / GYF domain-containing protein	3.376
AT3G52120	SWAP (Suppressor-of-White-APricot)/surp domain-containing protein / D111/G-patch domain-containing protein	3.376
AT4G02280	SUCROSE SYNTHASE 3 (SUS3)	3.375
AT5G18040	HEAT-INDUCED TAS1 TARGET 2 (HTT2)	3.373
AT1G15110	PHOSPHATIDYLSERINE SYNTHASE 1 (PSS1)	3.37
AT2G46150	Late embryogenesis abundant (LEA) hydroxyproline-rich glycoprotein family	3.369
AT3G07090	PPPDE putative thiol peptidase family protein	3.359
AT1G12580	PHOSPHOENOLPYRUVATE CARBOXYLASE-RELATED KINASE 1 (PEPKR1)	3.355
AT5G45710	ROOT HANDEDNESS 1 (RHA1)	3.355
AT2G45910	U-box domain-containing protein kinase family protein	3.353
AT2G29450	GLUTATHIONE S-TRANSFERASE TAU 5 (GSTU5)	3.352
AT4G13180	NAD(P)-binding Rossmann-fold superfamily protein	3.349
AT1G45145	THIOREDOXIN H-TYPE 5 (TRX5)	3.348
AT4G21940	CALCIUM-DEPENDENT PROTEIN KINASE 15 (CPK15)	3.345
AT4G34480	O-Glycosyl hydrolases family 17 protein	3.344
AT2G37710	L-TYPE LECTIN RECEPTOR KINASE IV.1 (LECRK-IV.1)	3.344

AT1G67310	Calmodulin-binding transcription activator protein with CG-1 and Ankyrin domains	3.343
AT5G64250	Aldolase-type TIM barrel family protein	3.342
AT2G04350	LONG-CHAIN ACYL-COA SYNTHETASE 8 (LACS8)	3.34
AT3G51440	Calcium-dependent phosphotriesterase superfamily protein	3.334
AT4G11800	Calcineurin-like metallo-phosphoesterase superfamily protein	3.33
AT5G04830	Nuclear transport factor 2 (NTF2) family protein	3.328
AT1G54100	ALDEHYDE DEHYDROGENASE 7B4 (ALDH7B4)	3.327
AT3G05580	TYPE ONE PROTEIN PHOSPHATASE 9 (TOPP9)	3.326
AT5G63130	Octicosapeptide/Phox/Bem1p family protein	3.325
AT2G02390	GLUTATHIONE S-TRANSFERASE ZETA 1 (GSTZ1)	3.322
AT3G58640	Mitogen activated protein kinase kinase kinase-related	3.321
AT3G02140	TWO OR MORE ABRES-CONTAINING GENE 2 (TMAC2)	3.319
AT3G44110	(J3)	3.314
AT5G26770	(ATNEAP2)	3.312
AT4G27350	Protein of unknown function (DUF1223)	3.307
AT3G19240	Vacuolar import/degradation, Vid27-related protein	3.303
AT5G65280	GCR2-LIKE 1 (GCL1)	3.301
AT4G31170	Protein kinase superfamily protein	3.298
AT4G28300	Encodes a protein with 13.6% proline amino acids that is predicted to localize to the cell wall. The mRNA is cell-to-cell mobile.	3.292
AT5G63790	NAC DOMAIN CONTAINING PROTEIN 102 (NAC102)	3.289
AT5G13160	AVRPPHB SUSCEPTIBLE 1 (PBS1)	3.288
AT2G33700	PROTEIN PHOSPHATASE 2C G GROUP 1 (PP2CG1)	3.285
AT2G30140	UDP-GLUCOSYL TRANSFERASE 87A2 (UGT87A2)	3.285
AT3G59770	SUPPRESSOR OF ACTIN 9 (SAC9)	3.284
AT3G28450	BAK1-INTERACTING RECEPTOR-LIKE KINASE 2 (BIR2)	3.282
AT4G22740	glycine-rich protein	3.281
AT4G11220	VIRB2-INTERACTING PROTEIN 2 (BTI2)	3.28
AT1G66880	Protein kinase superfamily protein	3.275
AT5G06750	ARABIDOPSIS PP2C CLADE D 8 (APD8)	3.274
AT5G02020	SALT INDUCED SERINE RICH (SIS)	3.274
AT5G41350	RING/U-box superfamily protein	3.266

AT1G16670	Protein kinase superfamily protein	3.265
AT2G45920	U-box domain-containing protein	3.263
AT3G10420	SEEDLING PLASTID DEVELOPMENT 1 (SPD1)	3.263
AT5G35460	unknown protein	3.26
AT3G46930	RAF-LIKE MITOGEN-ACTIVATED PROTEIN KINASE KINASE KINASE 43 (RAF43)	3.258
AT3G12350	F-box family protein	3.256
AT5G66620	DA1-RELATED PROTEIN 6 (DAR6)	3.255
AT3G63500	TITANIA 2 (TTA2)	3.252
AT3G59090	CANDIDATE G-PROTEIN COUPLED RECEPTOR 3 (CAND3)	3.251
AT5G25770	alpha/beta-Hydrolases superfamily protein	3.25
AT1G49050	(APCB1)	3.247
AT5G42810	INOSITOL-PENTAKISPHOSPHATE 2-KINASE 1 (IPK1)	3.242
AT1G69490	NAC-LIKE, ACTIVATED BY AP3/PI (NAP)	3.239
AT3G14200	Chaperone DnaJ-domain superfamily protein	3.236
AT2G45500	AAA-type ATPase family protein	3.234
AT4G12020	(WRKY19)	3.233
AT5G54940	Translation initiation factor SUI1 family protein	3.231
AT1G05340	unknown protein	3.229
AT2G01600	ENTH/ANTH/VHS superfamily protein	3.229
AT2G21820	unknown protein	3.228
AT5G12010	unknown protein	3.228
AT5G52070	Agenet domain-containing protein	3.228
AT4G27657	unknown protein	3.224
AT5G43100	Eukaryotic aspartyl protease family protein	3.221
AT5G61510	GroES-like zinc-binding alcohol dehydrogenase family protein	3.219
AT1G12360	KEULE (KEU)	3.216
AT2G30500	NETWORKED 4B (NET4B)	3.213
AT2G31990	Exostosin family protein	3.21
AT3G55960	Haloacid dehalogenase-like hydrolase (HAD) superfamily protein	3.21
AT1G66340	ETHYLENE RESPONSE 1 (ETR1)	3.208
AT3G15760	unknown protein	3.207

AT4G32160	Phox (PX) domain-containing protein	3.202
AT3G26000	RCAR3 INTERACTING F-BOX PROTEIN 1 (RIFP1)	3.194
AT4G11660	(AT-HSFB2B)	3.192
AT1G68440	unknown protein	3.191
AT4G33910	2-oxoglutarate (2OG) and Fe(II)-dependent oxygenase superfamily protein	3.189
AT4G33240	FORMS APLOID AND BINUCLEATE CELLS 1A (FAB1A)	3.186
AT4G32870	Polyketide cyclase/dehydrase and lipid transport superfamily protein	3.185
AT5G24120	SIGMA FACTOR E (SIGE)	3.182
AT4G18140	SCP1-LIKE SMALL PHOSPHATASE 4B (SSP4b)	3.178
AT3G03740	BTB-POZ AND MATH DOMAIN 4 (BPM4)	3.177
AT1G58360	AMINO ACID PERMEASE 1 (AAP1)	3.176
AT1G32700	PLATZ transcription factor family protein	3.175
AT4G05320	POLYUBIQUITIN 10 (UBQ10)	3.174
AT1G45976	S-RIBONUCLEASE BINDING PROTEIN 1 (SBP1)	3.174
AT3G12360	INCREASED TOLERANCE TO NACL (ITN1)	3.17
AT1G68300	Adenine nucleotide alpha hydrolases-like superfamily protein	3.169
AT2G38410	ENTH/VHS/GAT family protein	3.168
AT1G73080	PEP1 RECEPTOR 1 (PEPR1)	3.168
AT5G44090	Calcium-binding EF-hand family protein	3.165
AT2G42010	PHOSPHOLIPASE D BETA 1 (PLDBETA1)	3.165
AT4G22980	FUNCTIONS IN: molecular_function unknown	3.164
AT5G58940	CALMODULIN-BINDING RECEPTOR-LIKE CYTOPLASMIC KINASE 1 (CRCK1)	3.163
AT5G39660	CYCLING DOF FACTOR 2 (CDF2)	3.163
AT5G24590	TCV-INTERACTING PROTEIN (TIP)	3.162
AT5G01670	NAD(P)-linked oxidoreductase superfamily protein	3.158
AT5G04720	ADR1-LIKE 2 (ADR1-L2)	3.154
AT1G17230	Leucine-rich receptor-like protein kinase family protein	3.152
AT1G07630	POL-LIKE 5 (PLL5)	3.152
AT4G01070	(GT72B1)	3.15
AT2G17520	(IRE1A)	3.149
AT1G69270	RECEPTOR-LIKE PROTEIN KINASE 1 (RPK1)	3.149

AT3G51370	Protein phosphatase 2C family protein	3.148
AT2G27150	ABSCISIC ALDEHYDE OXIDASE 3 (AAO3)	3.148
AT4G19640	(ARA7)	3.147
AT2G46940	unknown protein	3.145
AT5G19240	Glycoprotein membrane precursor GPI-anchored	3.144
AT5G60360	ALEURAIN-LIKE PROTEASE (ALP)	3.144
AT5G07270	XB3 ORTHOLOG 3 IN ARABIDOPSIS THALIANA (XBAT33)	3.144
AT2G25520	Drug/metabolite transporter superfamily protein	3.143
AT4G02410	L-TYPE LECTIN RECEPTOR KINASE IV.3 (LECRK-IV.3)	3.137
AT1G77280	Protein kinase protein with adenine nucleotide alpha hydrolases-like domain	3.135
AT5G53970	TYROSINE AMINOTRANSFERASE 7 (TAT7)	3.135
AT4G22920	NON-YELLOWING 1 (NYE1)	3.134
AT3G51250	Senescence/dehydration-associated protein-related	3.133
AT3G44720	AROGENATE DEHYDRATASE 4 (ADT4)	3.133
AT3G51860	CATION EXCHANGER 3 (CAX3)	3.132
AT2G19710	IST1-LIKE 5 (ISTL5)	3.132
AT5G53220	unknown protein	3.131
AT4G24400	CBL-INTERACTING PROTEIN KINASE 8 (CIPK8)	3.13
AT1G50260	N-TERMINAL-TRANSMEMBRANE-C2 DOMAIN TYPE 5.1 (NTMC2T5.1)	3.126
AT4G15530	PYRUVATE ORTHOPHOSPHATE DIKINASE (PPDK)	3.123
AT1G71697	CHOLINE KINASE 1 (CK1)	3.12
AT1G61140	EMBRYO SAC DEVELOPMENT ARREST 16 (EDA16)	3.12
AT5G02620	ANKYRIN-LIKE1 (ANK1)	3.119
AT1G34750	Protein phosphatase 2C family protein	3.118
AT5G52580	RabGAP/TBC domain-containing protein	3.117
AT3G44630	Disease resistance protein (TIR-NBS-LRR class) family	3.116
AT3G14440	NINE-CIS-EPOXYCAROTENOID DIOXYGENASE 3 (NCED3)	3.115
AT4G02200	Drought-responsive family protein	3.113
AT3G51130	unknown protein	3.112
AT4G32020	unknown protein	3.112
AT3G46230	HEAT SHOCK PROTEIN 17.4 (HSP17.4)	3.104

AT1G31540	Disease resistance protein (TIR-NBS-LRR class) family	3.102
AT3G61630	CYTOKININ RESPONSE FACTOR 6 (CRF6)	3.101
AT5G54730	HOMOLOG OF YEAST AUTOPHAGY 18 (ATG18) F (G18F)	3.096
AT4G19140	unknown protein	3.095

Supplementary methods

Subtracted drought stress cDNA library construction in finger millet

For drought stress cDNA library construction, finger millet plants were grown in pots filled with mixture of red soil, sand and farmyard manure at 3:1:1 ratio in a rainout shelter facility under natural vapor pressure deficit of approximately 4 kPa and natural light conditions. Drought stress was applied on 25 days old plants by gradually withholding water and monitoring soil water status by gravimetric method [1-3]. Gravimetric method involves determination and regulation of soil moisture content on the basis of mass. Pots were filled with equal weight of soil and fresh weight (FW), saturated weight (SW) and dry weight (DW) were determined. FW of the soil was calculated as (FW of soil + pot weight)-(pot weight). SW of the soil was determined as (weight of soil flooded with water and drained overnight + pot weight)-(pot weight). DW of the soil was determined as (oven DW of soil + pot weight)-(pot weight). Soil moisture content at 100% field capacity (FC) was determined as [(FW-DW)/(SW-DW)] x 100. A batch of plants were maintained at 100% FC as control and soil moisture content for other drought stress levels was calculated based on 100% FC. Drought stress was applied by gradually reducing the soil water content to achieve desired stress levels by weighing the pots at 10 am every day. Sets of plants which reached 80% and 60% FC were maintained at that level by replenishing the water lost through evapotranspiration until plants designated for severe stress reached 35% FC. Further, plants at all stress levels were maintained at designated FC for 5 more days and leaf samples were collected. At the end of stress period, second and third fully expanded leaves were harvested from drought stressed and control plants, and used for cDNA library preparation and RT-qPCR analysis. Another set of drought stressed plants were re-watered and tissue was harvested at 6 h of recovery. Recovery samples were used only for RT-qPCR analysis.

Total RNA was isolated from control (100% FC) and drought stressed (80%, 60% and 35% FC) samples. Poly(A)+ RNA was purified by magnetic separation after annealing it to 5′-biotinylated oligo-dT(18) primer and subsequently immobilized onto streptavidin-linked paramagnetic beads. First-strand cDNA was synthesized from 5 μg of poly(A)+

RNA from control sample using 5′-biotinylated oligo-dT (T(18)VN) primer and SuperScript® II Reverse Transcriptase (Invitrogen Corporation, Carlsbad, CA, USA) following the manufacturer's protocol. One microgram of poly(A)+ RNA from pooled drought stress samples (tester) was mixed with 5-fold excess of 5′-biotinylated first-strand cDNA population of control sample (driver) and hybridized for 4 h at 68°C in 50 µl of RNase free hybridization buffer (10 mM Tris-HCl pH 7.5, 0.5 M KCl and 5 mM EDTA). The poly(A)+ RNA-cDNA hybrids and excess cDNA population were immobilized onto streptavidin beads. The beads were separated under magnetic field and the supernatant was transferred to a new tube. The unbound differentially expressed poly(A)+ RNA was precipitated with two volumes of absolute ethanol. The subtracted poly(A)+ RNA was used to synthesize double strand cDNA and uni-directionally ligated into Uni-ZAP XR vector, *in vitro* packaged and infected to XL1-Blue MRF *Escherichia coli* cells using a Uni-ZAP XR cDNA library construction kit (Stratagene, La Jolla, CA, USA), according to the manufacturer's instructions. The individual recombinant plaques from the cDNA library were transferred into tubes containing 200 µl of SM buffer (100 mM NaCl, 8 mM $MgSO_4.7H_2O$, 50 mM Tris-HCl pH 7, 0.04% gelatin). They were verified by PCR using M13 primers (Supplementary Table S1) with 1 µl of recombinant phage suspension as a template in 50 µl of PCR reaction mix (94°C for 1 min, 55°C for 1 min and 72°C for 2 min, for 30 cycles). PCR-amplified cDNA inserts were purified using PCR purification kit (Qiagen, Valencia, CA, USA) and used as template for sequencing. The sequences were annotated to assign putative functions via BLASTX non-redundant protein sequence database search. After removing the vector backbone sequence, the processed sequences were submitted to dbEST database at NCBI (Supplementary Table S3).

Gene expression analysis in finger millet

Total RNA was extracted from leaf tissue using RNeasy Plant Mini Kit (QIAGEN Inc. Valencia, CA, USA). First-strand cDNA was synthesized from 2 µg of total RNA with SuperScript^{TM} III first-strand synthesis system (Invitrogen^{TM} life technologies, Grand Island, NY, USA) and used as a template for RT-qPCR and semi-quantitative RT-PCR analysis. Equal dilution of cDNA was used for RT-qPCR analysis with gene specific

primers (Supplementary Table S1). RT-qPCR was performed using SYBR® Green real-time PCR master mix (Invitrogen™ life technologies, Grand Island, NY, USA) on Real-Time PCR detection system (Bio-Rad Life Science Research, Hercules, CA, USA) as per the manufacturer recommended protocol. The experiment was repeated using different batch of cDNA preparation in another machine (ABI PRISM 5700, Applied Biosystems, Bedford, MA, USA). The average Ct values of two biological replicates and three technical replicates were used for analysis. The data was normalized with the *EcActin1* (GenBank accession number HE800188) and fold change in expression under a given drought stress level compared to control was calculated using the $2^{-\Delta\Delta Ct}$ method [4,5].

Full-length cloning of *EcGBF3*

RNA ligase-mediated rapid amplification of cDNA ends (RLM-RACE) was performed using the GeneRacer kit (Invitrogen Corporation, Carlsbad, CA, USA) according to the manufacturer's instructions to isolate 5′ and 3′ regions of truncated *EcGBF3* gene from finger millet. The 5′ region was amplified using the 5′ GeneRacer primer and gene specific reverse primer, and 3′ region was amplified using the 3′ GeneRacer primer and forward gene specific primer which were designed to leave an overlapping sequence at both 5′ and 3′ ends of truncated *EcGBF3* gene (Supplementary Table S1). Full-length *EcGBF3* gene was confirmed by sequencing and alignment to the orthologous sequences from other plant species.

Abiotic stress assays on MS medium

One week old hygromycin resistant T_3 generation plants from three lines per construct were used for stress experiments. Seedlings were transferred on to half strength MS medium (0.5% sucrose and 0.1% phytagel) supplemented with 100 mM mannitol, 100 mM NaCl or 3 μM ABA. Instead of agar, lower concentration of phytagel was used as a solidifying agent for easy uprooting of seedlings from the plates. Plants were carefully uprooted from the media, blotted on paper towel to remove any media and used for root length and biomass measurements. Student's *t* test was used to assess the statistical

significance of the differences in the means of *Atgbf3* mutant or transgenic plants from Col-0 plants.

References

1 Ramegowda, V. *et al.* Expression of a finger millet transcription factor, EcNAC1, in tobacco confers abiotic stress-tolerance. *PLoS One* **7**, e40397, doi:10.1371/journal.pone.0040397 (2012).

2 Karaba, A. *et al.* Improvement of water use efficiency in rice by expression of HARDY, an Arabidopsis drought and salt tolerance gene. *Proceedings of the National Academy of Sciences* **104**, 15270-15275, doi:10.1073/pnas.0707294104 (2007).

3 Kathuria, H. *et al.* Glycinebetaine-induced water-stress tolerance in codA-expressing transgenic indica rice is associated with up-regulation of several stress responsive genes. *Plant biotechnology journal* **7**, 512-526 (2009).

4 Livak, K. J. & Schmittgen, T. D. Analysis of Relative Gene Expression Data Using Real-Time Quantitative PCR and the 2−ΔΔCT Method. *Methods* **25**, 402-408, doi:http://dx.doi.org/10.1006/meth.2001.1262 (2001).

5 Schmittgen, T. D. & Livak, K. J. Analyzing real-time PCR data by the comparative CT method. *Nature Protocols* **3**, 1101-1108 (2008).
